# Body weight influences musculoskeletal adaptation to long-term voluntary wheel running during aging in female mice

**DOI:** 10.18632/aging.204390

**Published:** 2022-11-18

**Authors:** Yukiko Kitase, Julian A. Vallejo, Sarah L. Dallas, Yixia Xie, Mark Dallas, LeAnn Tiede-Lewis, David Moore, Anthony Meljanac, Corrine Kumar, Carrie Zhao, Jennifer Rosser, Marco Brotto, Mark L. Johnson, Ziyue Liu, Michael J. Wacker, Lynda Bonewald

**Affiliations:** 1 Department of Anatomy, Cell Biology and Physiology, School of Medicine, Indiana University, Indianapolis, IN 46202, USA; 2 Department of Oral and Craniofacial Sciences, School of Dentistry, University of Missouri, Kansas City, MO 64108, USA; 3 Bone-Muscle Research Center, College of Nursing and Health Innovation, University of Texas, Arlington, TX 76019, USA; 4 Department of Biostatistics and Health Data Science, School of Medicine, Indiana University, Indianapolis, IN 46202, USA; 5 Department of Biomedical Sciences, School of Medicine, University of Missouri, Kansas City, MO 64108, USA; 6 Department of Orthopaedic Surgery, School of Medicine, Indiana University, Indianapolis, IN 46202, USA

**Keywords:** body weight, musculoskeletal adaptation, long-term, voluntary wheel running, aging

## Abstract

Frailty is the hallmark of aging that can be delayed with exercise. The present studies were initiated based on the hypothesis that long-term voluntary wheel running (VWR) in female mice from 12 to 18 or 22 months of age would have beneficial effects on the musculoskeletal system. Mice were separated into high (HBW) and low (LBW) body weight based on final body weights upon termination of experiments. Bone marrow fat was significantly higher in HBW than LBW under sedentary conditions, but not with VWR. HBW was more protective for soleus size and function than LBW under sedentary conditions, however VWR increased soleus size and function regardless of body weight. VWR plus HBW was more protective against muscle loss with aging. Similar effects of VWR plus HBW were observed with the extensor digitorum longus, EDL, however, LBW with VWR was beneficial in improving EDL fatigue resistance in 18 mo mice and was more beneficial with regards to muscle production of bone protective factors. VWR plus HBW maintained bone in aged animals. In summary, HBW had a more beneficial effect on muscle and bone with aging especially in combination with exercise. These effects were independent of bone marrow fat, suggesting that intrinsic musculoskeletal adaptions were responsible for these beneficial effects.

## INTRODUCTION

Aging increases the prevalence of sarcopenia and osteoporosis that are often both components of a musculoskeletal syndrome, osteosarcopenia. Osteoporosis or bone loss with aging, is a disease of not only reduced bone density but also bone quality and is associated with high fracture risk. Hip fracture can be a catastrophic event as 20–30% of elderly patients die within one year of experiencing a hip fracture [1]. Hip fractures are also associated with loss of mobility, loss of independence and the need for institutionalized care. Osteosarcopenia is highly associated with frailty, falls, fractures and disability leading to decreased quality of life and increased morbidity and mortality [2–5]. Moreover, it is a major public health issue accompanied by a significant burden of medical costs in aging societies [6]. The U.S. Census Bureau anticipates that for the first time people aged 65 years and older will outnumber those less than 18 years old by the year 2034 (https://www.census.gov/library/stories/2018/03/graying-america.html). The aging process results in the functional decline of many organ systems including bone and skeletal muscle. Thus, disease prevalence within the population and the resulting economic burden will also continue to rise in the coming decades.

Of particular concern is the gradual deterioration in skeletal muscle tissue mass and functional capacity beginning at middle-age and progressing into old age, known clinically as sarcopenia [7]. There is a significant public health burden associated with sarcopenia given a prevalence of up to 35% in elderly adults [8] and accounting for an estimated 18.5 billion dollars per year in health care related costs in the United States alone [9]. At an individual level, the reduced physical capability of muscle experienced during sarcopenia leads to a heightened risk of the development of additional disability and mortality through an increased chance of falls and related injuries such as fractures [10]. Accordingly, muscle decline is considered a major burden of disease. The etiology of muscle loss and weakness with aging is due to a multitude of underlying factors that affect both the quantity and the quality of muscle. These include muscle fiber atrophy [11], infiltration of non-muscle tissue [12], neuromuscular unit loss [13], reduced oxidative metabolism [14] and altered excitation-contraction coupling mechanisms [15, 16].

The consequences of aging are compounded by lack of exercise as physical inactivity is a primary factor contributing to the prevalence of sarcopenia and bone loss in the aging population. Exercise maintains the health of the musculoskeletal system, the system most likely responsible for the health of the individual. The musculoskeletal system is responsible for the beneficial effects of mobility on brain function, cardiac function, metabolism, and other organs [17]. Exercise appears to delay the negative effects of aging not only on the musculoskeletal system but also on other diseases associated with aging such as dementia, Amyotrophic Lateral Sclerosis, and Alzheimer’s. Skeletal muscle is known to function as an endocrine organ and release hormones and metabolites, especially during periods of heightened activity, which may mediate the beneficial health effects of exercise on other organs, including bone [18, 19].

Regular exercise using either aerobic or resistance modalities has been shown to be effective in improving the aging-related decline in muscle mass and performance, with no other treatment strategies to date proving superior [20]. However, exercise interventions are typically implemented subsequent to the manifestation of disease. The World Health Organization recently published a report that places a substantial emphasis on maintaining adequate functional ability throughout the aging process in order to help support a healthy aging lifestyle [21]. This represents a transformation in the current treatment paradigm for aging from a disease-centered reactionary approach to a more proactive function-centered strategy.

Clearly physical activity can have a positive impact on the skeleton with aging [22, 23]. Whereas low impact aerobic exercises such as swimming or biking have little effect on bone mass, high-impact exercises, such as brisk walking, running and jumping, are most commonly recommended to prevent or treat osteoporosis as are resistance training where muscles are being contracted [24]. Resistance training is one of the most effective non-pharmacological means to increase bone mineral density [24]. The positive effects of resistance exercise on bone are thought to be mediated through the osteocyte, the mechanosensory cell in bone [25].

Frequently osteoporosis and sarcopenia occur concurrently. It is not known if one precedes the other or if one condition influences disease progression of the other condition [26, 27]. We hypothesized that long-term voluntary exercise started later in life (12 months of age) would improve both skeletal muscle and bone parameters in aging female mice up to 22 months. We chose a voluntary exercise program (for 6 mo and 10 mo) without the use of resistance for female mice beginning at middle age to determine the impact of maintaining fast and slow skeletal muscle mass and contractility and bone mass and properties into old age.

## RESULTS

### Effects of voluntary wheel running on body weight, bone marrow adipose tissue, and heart weight

**Figure 1:**
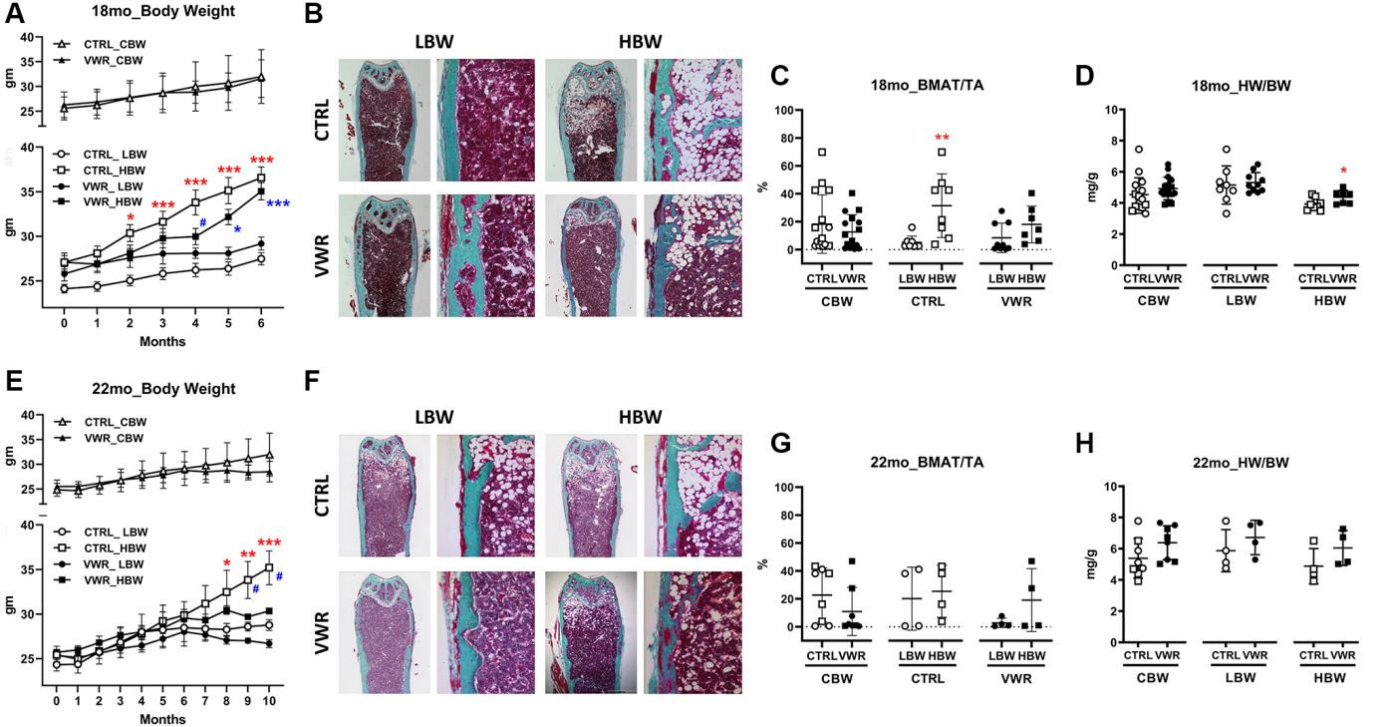
**Body weight, bone marrow adipose tissue, and heart weight during aging.** The low body weight, LBW, and high body weight, HBW, groups showed differences in bone marrow adipose tissue. 6 mo endurance exercise increased heart weight in the HBW subgroup. (**A**) The body weight of 18 mo. ^*^: *p* < 0.05 and ^***^: *p* < 0.001, CTRL/HBW vs. CTRL/LBW mice (red asterisks). ^*^: *p* < 0.05 and ^***^: *p* < 0.001, VWR/HBW vs. VWR/LBW mice (blue asterisks). ^#^: *p* < 0.05, VWR/HBW vs. CTRL/HBW mice. (**B** and **C**) Bone marrow adipose tissue/total area (BMAT/TA) ratio in 18 mo. ^**^: *p* < 0.01, CTRL/HBW vs. CTRL/LBW mice. (**D**) Heart muscle weight/body weight (HW/BW) ratio in 18 mo. ^*^: *p* < 0.05, CTRL/HBW vs. VWR/HBW mice. (**E**) The body weight of 22 mo. ^*^: *p* < 0.05, ^**^: *p* < 0.01, and ^***^: *p* < 0.001, CTRL/HBW vs. CTRL/LBW mice. ^#^: *p* < 0.05, VWR/HBW vs. CTRL/HBW mice. (**F** and **G**) Bone marrow adipose tissue/total area (BMAT/TA) ratio in 22 mo. (**H**) Heart muscle weight/body weight (HW/BW) ratio in 22 mo (**D**). Abbreviations: CTRL: control group; VWR: voluntary wheel running group; CBW: Combined groups; LBW: Low body weight group; HBW: High body weight group.

As large variability was observed in the final body weights (Minimum: 24.6 and 25.6 g, Maximum: 41.9 and 40.2 g for 18 and 22 mo cohorts, respectively (Table 1), the mice were divided into low body weight (LBW) and high body weight (HBW) subgroups. This separation was based on the fact that the large mice would be characterized as obese [28] and because fat mass is intimately related to skeletal muscle function and bone mass/bone mineral density (BMD) [29–31] which could have a bearing on interpretation of results. The subgroups were defined as being below (LBW) and above (HBW) the median of the final body weights for each group, giving a sample size of 8–10 for the LBW and HBW subgroups for the 18 mo old mice and 4 for the 22 mo mice. The average final weight in the HBW subgroup was significantly higher than the LBW subgroup in all groups except for the 2 mo VWR/HBW mice. VWR in the 22 mo old mice resulted in significantly decreased final body weight in the HBW group compared to CTRL, resulting in no difference between LBW and HBW subgroups (Table 1). All groups gained weight over the study period (Figure 1A and 1E). HBW and LBW subgroups in CTRL mice began to diverge significantly from each other at around 2–4 months before the end of the study. Correlations were observed between final body weight and a number of bone and muscle parameters (Supplementary Table 1). Final body weight was positively correlated with cortical bone parameters but negatively correlated with fast-twitch skeletal muscle mass and fatigability in 18 mo old mice. In contrast, final body weight was positively associated with trabecular bone parameters, skeletal muscle protective factors, and fast-twitch contractile functions in 22 mo old mice.

**Table 1:** The profile of body weight in LBW/HBW mice with or without long-term endurance exercise.

**GROUP**	**18 mo old**		**22 mo old**	
	**CTRL**	**VWR**	**CTRL**	**VWR**
**CBW**	*N* = 16	*N* = 17	*N* = 8	*N* = 8
**LBW**	*N* = 8	*N* = 10	*N* = 4	*N* = 4
**HBW**	*N* = 8	*N* = 7	*N* = 4	*N* = 4
**INITIAL BODY WEIGHT (gm)**				
**CBW**	25.6 ± 2.3	26.3 ± 2.6	24.9 ± 1.3	25.5 ± 1.3
	(22.3–31.1)	(22.9–30.8)	(22.6–27.1)	(24.00–27.50)
**LBW**	24.1 ± 1.3	25.8 ± 2.5	24.3 ± 1.4	25.4 ± 1.4
	(22.3–25.6)	(22.9–29.8)	(22.6–26.0)	(24.00–27.10)
**HBW**	27.1 ± 2.5	27.1 ± 2.7	25.5 ± 1.1	25.7 ± 1.4
	(23.6–31.1)	(23.0–30.8)	(24.4–27.1)	(24.2–27.5)
**FINAL BODY WEIGHT (gm)**				
**CBW**	32.0 ± 5.4	31.6 ± 3.8	32.0 ± 4.3	28.5 ± 2.1
	(25.5–41.9)	(24.6–39.5)	(27.1–40.2)	(25.6–31.1)
**LBW**	27.5 ± 1.9	29.2 ± 2.3	28.8 ± 1.2	26.7 ± 0.9
	(25.5–31.1)	(24.6–32.1)	(27.1–29.0)	(25.6–27.7)
**HBW**	36.6 ± 3.4^b^	35.1 ± 2.6^b^	35.2 ± 3.8 ^b^	30.4 ± 0.7^a^
	(32.4–41.9)	(32.5–39.5)	(32.1–40.2)	(29.5–31.1)
**BODY WEIGHT CHANGE (%)**				
**CBW**	24.7 ± 14.8	20.8 ± 16.2	28.4 ± 15.5	11.9 ± 9.8^a^
	(7.5–56.6)	(−4.70–53.10)	(14.6–57.7)	(−5.6–28.5)
**LBW**	14.0 ± 7.2	13.9 ± 12.2	18.3 ± 2.5	5.6 ± 7.8^a^
	(7.5–29.0)	(−4.7–33.0)	(14.6–19.9)	(−5.6–12.7)
**HBW**	**35.5 ± 12.3^b^**	**30.6 ± 16.8^b^**	38.6 ± 16.6	18.3 ± 7.4
	(20.0–56.6)	(12.3–53.1)	(18.9–57.7)	(10.9–28.5)

Data are mean ± SD (range). Abbreviations: CTRL: control group; VWR: voluntary wheel running group; CBW: Combined groups; LBW: Low body weight group; HBW: High body weight group. ^a^*p* < 0.05 vs. Corresponding CTRL, ^b^*p* < 0.05 vs. Corresponding LBW.

As aging/weight gain/obesity is positively associated with the expansion of bone marrow adipose tissue (BMAT) (Review by [32]), this parameter was also quantified (Figure 1B, 1C, 1F and 1G). 18 mo CTRL/HBW mice showed higher BMAT compared to CTRL/LBW mice, but the difference was lost with 6 mo of VWR. There were no significant differences found in 22 mo old mice most likely due to the high variability with a small sample number.

The weekly running distance for the 18 and 22 mo old VWR mice are shown in Supplementary Figure 1A and 1B. There were no significant differences in running distance between LBW and HBW mice at either age. The average running distance was 4.2 and 4.8 km/day with a peak distance of 6.5 and 8.1 km/day for 18 and 22 mo old mice, respectively. The distance run gradually decreased with time and age and became half of the peak distance at 15 and 24 wks. The average running distance was reduced to 2.6 and 2.2 km/day during the last month, for 18 and 22 mo old VWR mice, respectively.

Endurance exercise is associated with increases in heart mass or physiological cardiac hypertrophy in both humans and in rodent models of exercise [33–36]. To determine the effect of this long-term endurance exercise model on heart size with aging, we measured heart weight (HW) and heart weight normalized to body weight (HW/BW). Absolute heart weights were significantly larger in 18 mo mice with VWR in the combined body weight, CBW, (CTRL: 142.1 ± 23.2 mg and VWR: 154.0 ± 16.4 mg; CBW, *p* < 0.05, data not shown). As seen in Figure 1D and 1H, the HBW subgroup in 18 mo old VWR mice exhibited a significant 13% increase in HW/BW over CTRL (HBW*, p* < 0.05). No significant difference in heart size was observed in the 22 mo old sedentary or wheel running animals.

Aging induces pathological alterations including changes to cardiac myosin heavy chain (*Myhc*) isoform expression towards fetal *beta-Myhc* [37, 38], which could be mitigated by exercise [39, 40]. To determine the effect of our long-term endurance exercise model on heart MHC expression with aging, we evaluated the ratio of mRNA expression levels of *alpha-Myhc* relative to *beta-Myhc* in heart tissue from CTRL and VWR mice. No significant differences were found in *alpha-Myhc/beta-Myhc* expression ratio among any of the groups analyzed (Data not shown).

### The effects of VWR, body weight, and aging on soleus and EDL muscle mass and contractile force

**Figure 2:**
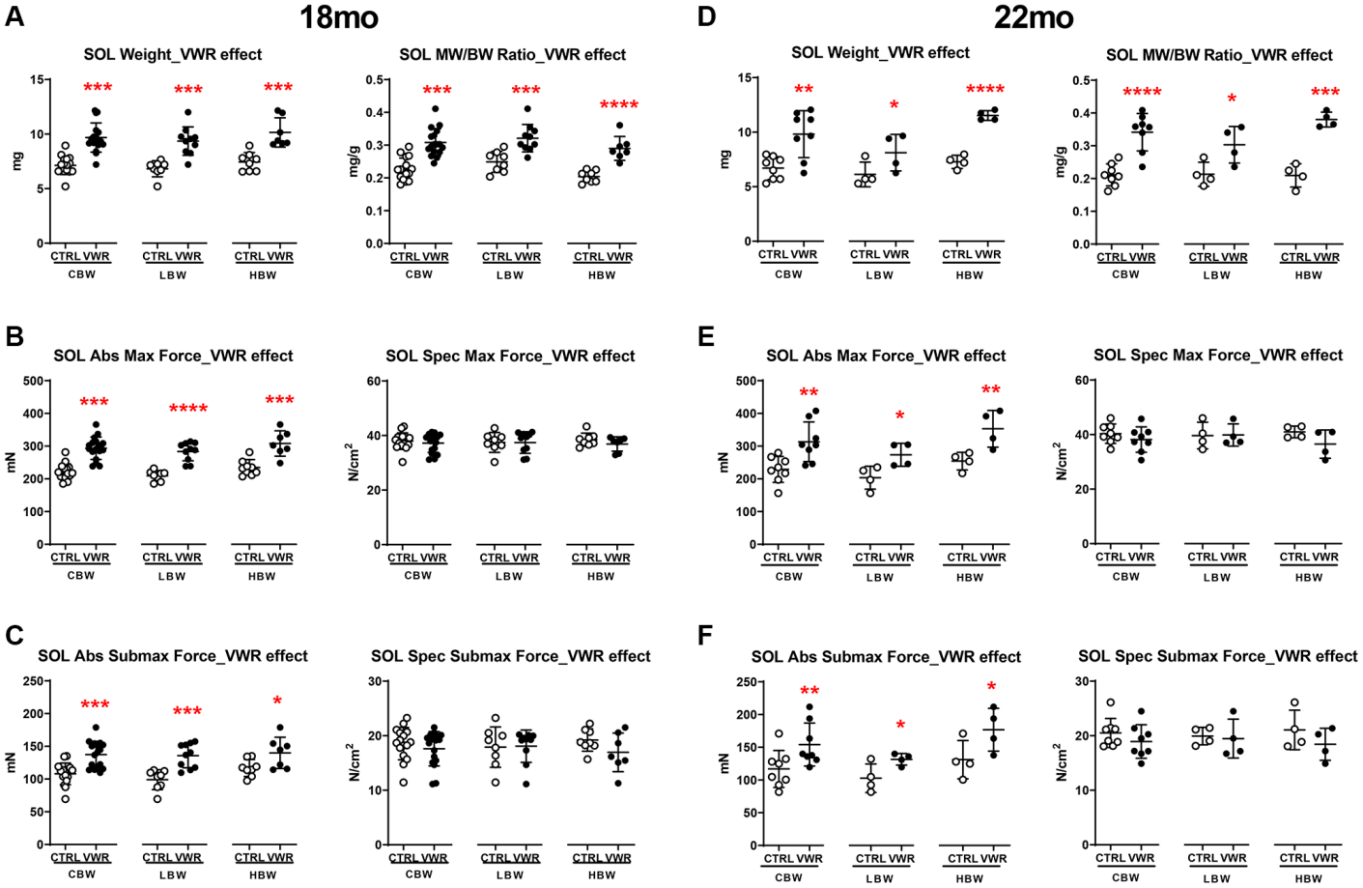
**The effect of long-term endurance exercise on soleus skeletal muscle weights and contractile force.** Endurance exercise resulted in hypertrophy of soleus muscles in all body weight groups in both 18 mo and 22 mo old mice. Endurance exercise also increased absolute force output in all body weight groups, while force normalized to muscle size was not changed in both 18 mo and 22 mo old mice. (**A**) Soleus muscle weight (left) and muscle weight/body weight (MW/BW) ratio (right) for 18 mo mice. (**B**) Soleus muscle absolute maximal force (left) and specific maximal force (right) in 18 mo mice. (**C**) Soleus muscle absolute submaximal force (left) and specific submaximal force (right) in 18 mo mice. (**D**) Soleus muscle weight (left) and muscle weight/body weight (MW/BW) ratio (right) for 22 mo mice. (**E**) Soleus muscle absolute maximal force (left) and specific maximal force (right) in 22 mo mice. (**F**) Soleus muscle absolute submaximal force (left) and specific submaximal force (right) in 22 mo mice. ^*^: *p* < 0.05, ^**^: *p* < 0.01, ^***^: *p* < 0.001, and ^****^: *p* < 0.0001. VWR vs. CTRL mice. Abbreviations: CTRL: control group; VWR: voluntary wheel running group; CBW: Combined groups (18 mo: *n* = 16 CTRL, *n* = 17 VWR; 22 mo: *n* = 8 CTRL, *n* = 8 VWR); LBW, Low body weight group (18 mo: *n* = 8 CTRL, *n* = 10 VWR; 22 mo: *n* = 4 CTRL, *n* = 4 VWR); HBW, High body weight group (18 mo: *n* = 8 CTRL, *n* = 7 VWR; 22 mo: *n* = 4 CTRL, *n* = 4 VWR).

Aging is associated with a reduction in muscle mass and performance. To determine the effects of long-term VWR on the maintenance of skeletal muscle tissue mass and contractility, we isolated soleus and EDL muscles after long-term VWR in 18 mo and 22 mo mice. In the slow-twitch soleus muscle, VWR exercise induced marked hypertrophy (Figure 2A and 2D, *p* < 0.05), greater force of contraction (Figure 2B and 2E, *p* < 0.05) and faster rates of force development and relaxation at both 18 and 22 mo (Supplementary Figure 2A–2D, *p* < 0.05) regardless of body weight. Since strength of muscle contraction can be related to size of the muscle (including myofiber number and cross-sectional area) or size independent alterations in excitation-contraction coupling, we normalized force to the physiological cross-sectional area of the muscle, which takes into account muscle weight, length and density when calculating force. There were no differences in the soleus specific contractile forces among sedentary and VWR mice of all body weight groups (Figure 2B, 2C and 2E, 2F, *p* > 0.05), indicating that the gains in absolute strength with VWR were proportional to the increases in muscle mass.

**Figure 3:**
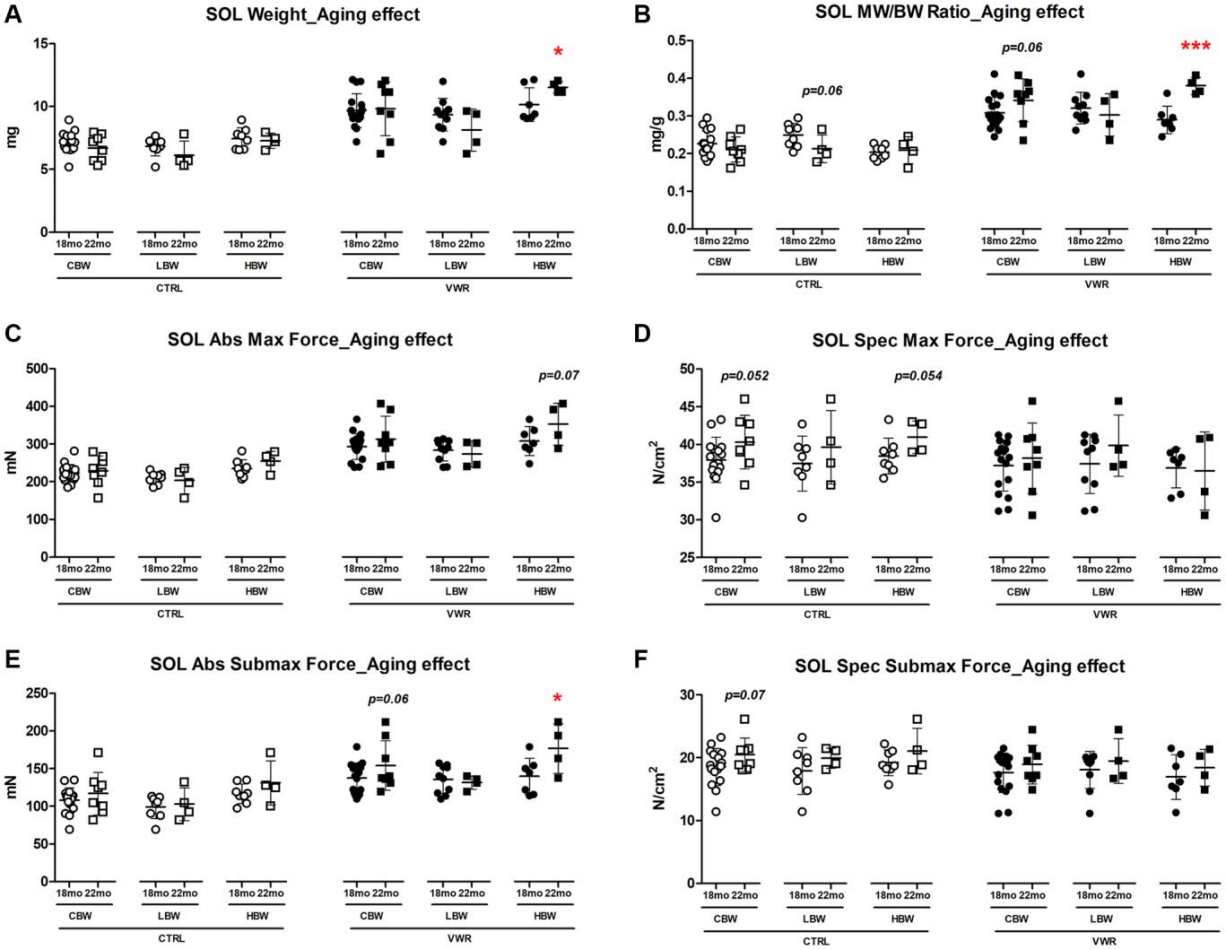
**Aging effects on soleus skeletal muscle weights and contractile force.** Long-term endurance exercise increased soleus skeletal muscle weight and contractile force during aging from 18 mo to 22 mo in HBW, but not LBW mice. (**A**) Soleus muscle weight, (**B**) muscle weight/body weight (MW/BW) ratio, (**C**) absolute maximal contractile force, (**D**) specific maximal contractile force, (**E**) absolute submaximal contractile force and (**F**) specific submaximal contractile force in 18 mo and 22 mo mice. ^*^: *p* < 0.05, ^***^: *p* < 0.001. VWR vs. CTRL mice. Abbreviations: CTRL: control group; VWR: voluntary wheel running group; CBW: Combined groups (18 mo: *n* = 16 CTRL, *n* = 17 VWR; 22 mo: *n* = 8 CTRL, *n* = 8 VWR); LBW: Low body weight group (18 mo: *n* = 8 CTRL, *n* = 10 VWR; 22 mo: *n* = 4 CTRL, *n* = 4 VWR); HBW: High body weight group (18 mo: *n* = 8 CTRL, *n* = 7 VWR; 22 mo: *n* = 4 CTRL, *n* = 4 VWR).

We next examined the specific effect of aging on the soleus muscle. When considering the effect of aging from 18 to 22 mo on soleus muscle size and contractile strength, there were no changes detected in CTRL mice regardless of body weight grouping (Figure 3 and Supplementary Figure 3, *p* > 0.05). However, when combined with VWR, having HBW maintained soleus muscle hypertrophy with aging as demonstrated by significantly increased muscle weight and contractile force from 18 mo to 22 mo, whereas no additional hypertrophy occurred in soleus muscles from LBW mice performing VWR during this period of aging (Figure 3A, 3B, 3E and Supplementary Figure 3A, 3B, *p* < 0.05).

**Figure 4:**
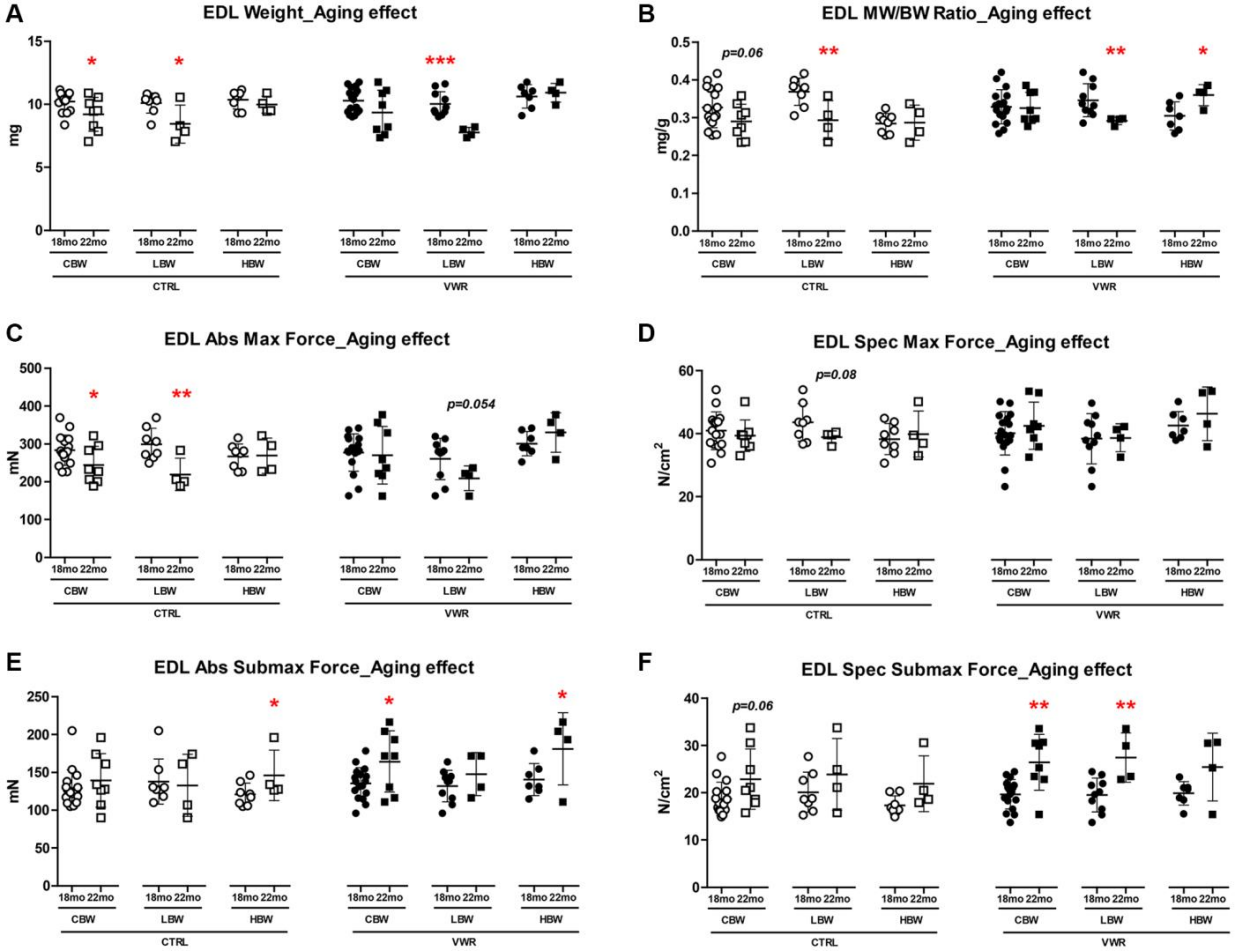
**Aging effects on EDL skeletal muscle weights and contractile force.** Long-term endurance exercise protected against EDL skeletal muscle atrophy and contractile force loss during aging from 18 mo to 22 mo. HBW but not LBW, without exercise also protected against aging-related declines in these parameters in EDL muscle. (**A**) EDL muscle weight, (**B**) muscle weight/body weight (MW/BW) ratio, (**C**) absolute maximal contractile force, (**D**) specific maximal contractile force, (**E**) absolute submaximal contractile force and (**F**) specific submaximal contractile force in 18 mo and 22 mo LBW and HBW mice. ^*^: *p* < 0.05, ^**^: *p* < 0.01, ^***^: *p* < 0.001. VWR vs. CTRL mice. Abbreviations: CTRL: control group; VWR: voluntary wheel running group; CBW: Combined groups (18 mo: *n* = 16 CTRL, *n* = 17 VWR; 22 mo: *n* = 8 CTRL, *n* = 8 VWR); LBW: Low body weight group (18 mo: *n* = 8 CTRL, *n* = 10 VWR; 22 mo: *n* = 4 CTRL, *n* = 4 VWR); HBW: High body weight group (18 mo: *n* = 8 CTRL, *n* = 7 VWR; 22 mo: *n* = 4 CTRL, *n* = 4 VWR).

The primarily fast-twitch EDL muscle showed fewer overall alterations in muscle mass and strength when considering the effect of VWR alone (Supplementary Figure 4A, 4E and 4F). Interestingly, increases in EDL muscle weight/body weight ratio (Supplementary Figure 4D, HBW, *p* < 0.05), absolute and specific forces (Supplementary Figure 4B, 4C, HBW, *p* < 0.05), and rates of force development and relaxation (Supplementary Figure 2E–2H, HBW, *p* < 0.05) with VWR were detected only in the HBW group at 18 mo. Similarly, gastrocnemius muscle weight/body weight ratio was greater in 22 mo old VWR mice (CTRL: 3.66 ± 0.243 and VWR: 3.96 ± 0.202; CBW, *p* < 0.01, Data not shown), which when separated by body weight was found to be due to an increase in the HBW group and not the LBW group (CTRL: 3.50 ± 0.204 and VWR: 3.99 ± 0.149; HBW, *p* < 0.01, Data not shown). Upon further examination of the effect of aging from 18 mo to 22 mo we found a significant decrease in EDL muscle size, contractile force and contractile kinetics in sedentary CTRL mice which were attributed to reductions in the LBW group specifically while the HBW group was resistant to these age-related changes (Figure 4A–4C and Supplementary Figure 3C, *p* < 0.05). Long-term VWR was associated with increases in EDL submaximal forces and largely eliminated the declines seen in EDL muscle contractile strength and kinetic parameters from 18 to 22 mo (Figure 4A, 4C–4F and Supplementary Figure 3C, *p* < 0.05).

**Figure 5:**
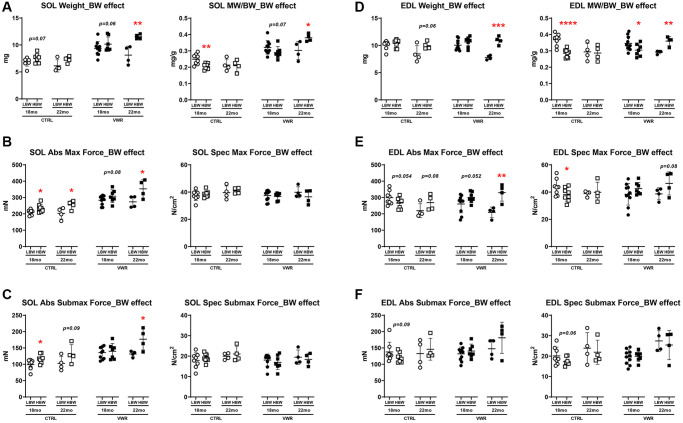
**Body weight effects on soleus and EDL skeletal muscle weights and contractile force.** (**A**) Soleus muscle weight (left) and muscle weight/body weight (MW/BW) ratio (right), (**B**) absolute maximal contractile force (left) and specific maximal contractile force (right), (**C**) absolute submaximal contractile force (left) and specific submaximal contractile force (right) in LBW and HBW mice. (**D**) EDL muscle weight (left) and muscle weight/body weight (MW/BW) ratio (right), (**E**) absolute maximal contractile force (left) and specific maximal contractile force (right), (**F**) absolute submaximal contractile force (left) and specific submaximal contractile force (right) in LBW and HBW mice. ^*^: *p* < 0.05, ^**^: *p* < 0.01, ^***^: *p* < 0.001 and ^****^: *p* < 0.0001. VWR vs. CTRL mice. Abbreviations: CTRL: control group; VWR: voluntary wheel running group; LBW: Low body weight group (18 mo: *n* = 8 CTRL, *n* = 10 VWR; 22 mo: *n* = 4 CTRL, *n* = 4 VWR); HBW: High body weight group (18 mo: *n* = 8 CTRL, *n* = 7 VWR; 22 mo: *n* = 4 CTRL, *n* = 4 VWR).

With regard to the effect of body weight on soleus muscle properties, we found having a heavier final body weight alone positively influenced soleus muscle contractile function in CTRL mice with the HBW group showing significantly augmented absolute contractile force and kinetics of contraction than the LBW group at both 18 and 22 mo timepoints (Figure 5B, 5C and Supplementary Figure 5A, 5B, *p* < 0.05). After long-term VWR, the HBW group exhibited larger soleus muscle weight, muscle weight/body weight ratio, absolute contractile force and faster kinetics of contraction compared to LBW mice, however, this effect was only observed at 22 mo of age and not 18 mo (Figure 5A–5C and Supplementary Figure 5A, 5B, *p* < 0.05). Upon examination of the direct effect of final body weight on EDL muscle parameters we found that under sedentary conditions, EDL muscles from the HBW group had reduced muscle weight/body weight ratio and specific contractile force compared to the LBW group at 18 mo (Figure 5D–5F, *p* < 0.05). On the other hand, the HBW group that underwent VWR until 22 mo of age had significantly improved muscle weight, muscle weight/body weight ratio, absolute contractile force and kinetics of contraction when compared against age-matched VWR mice in the LBW group (Figure 5D, 5E and Supplementary Figure 5C and 5D, *p* < 0.05).

In light of these findings suggesting body weight as an important modulator of soleus and EDL muscle size and function during exercise and aging, we utilized multivariable regression analysis with three-way interactions to test for interaction effects among body weight grouping, VWR, and aging on muscle parameters. For soleus muscle, the analysis detected a significant two-way interaction between body weight and age for muscle size and contractile function parameters (Table 2, *p* < 0.05). Similar to our initial results, HBW mice were determined to have significantly greater soleus muscle weight (*p* = 0.0007), contractile force output (*p* = 0.0005–0.0025) and kinetic parameters of contraction (*p* < 0.0001–0.0008) compared to LBW mice, and HBW was associated with an increase in these parameters during aging from 18 mo to 22 mo (*p* < 0.0001–0.025). In EDL muscle, regression analysis showed two-way interactions between body weight and VWR and aging, which was also in line with our initial analyses (Table 2, *p* < 0.05). There was a significant interaction between body weight and VWR for EDL muscle size and contractile function with VWR showing positive effects on force production (*p* = 0.0002) and rates of force development (*p* = 0.0004) and relaxation (*p* = 0.0001) in the HBW group but not LBW group. Lastly, body weight was found to interact with aging leading to reductions in EDL muscle size (*p* < 0.0001–0.0009), contractile force (*p* = 0.0034) and rate of force relaxation (*p* = 0.0099) from 18 mo to 22 mo in LBW mice while HBW mice were protected from these age-related declines (*p* > 0.05).

**Table 2:** Interaction analysis by multivariable regression on skeletal muscle and bone parameters.

**Soleus muscle parameters**	**Significant interactions**	**Statistics**	
		***F***	***P***
Muscle weight	BW*AGE	5.18	0.0278
Muscle weight/body weight	VWR*AGE	5.28	0.0265
	BW*AGE	10.94	0.0019
Absolute max force	BW*AGE	4.44	0.0408
Absolute submax force	BW*AGE	4.4	0.0418
Rate of force development (max force)	BW*AGE	15.43	0.0003
Rate of force development (submax force)	BW*AGE	10.48	0.0023
Rate of relaxation (max force)	BW*AGE	5.82	0.0201
Rate of relaxation (submax force)	BW*AGE	6.04	0.018
Calcium depletion (max force)	BW*VWR*AGE	6.01	0.0186
Calcium depletion (submax force)	BW*VWR*AGE	8.41	0.006
Recovery from calcium depletion (max force)	BW*VWR*AGE	5.57	0.0231
5 min of fatigue (submax) force	BW*AGE	11.93	0.0229
**EDL muscle parameters**	**Significant interactions**	**Statistics**	
		***F***	***P***
Muscle weight	BW*AGE	12.26	0.0011
Muscle weight/body weight	BW*VWR	6.38	0.0153
	BW*AGE	16.14	0.0002
Absolute max force	BW*VWR	8.91	0.0047
	BW*AGE	9.56	0.0035
Specific max force	BW*VWR	6.07	0.0177
Rate of force development (max force)	BW*VWR	5.55	0.0231
	BW*AGE	10.15	0.0027
Rate of force development (submax force)	BW*AGE	5.11	0.0287
Rate of relaxation (max force)	BW*VWR	8.4	0.0059
	BW*AGE	10.48	0.0023
Recovery from calcium depletion (submax force)	BW*AGE	4.78	0.0345
**Cortical bone parameters**	**Significant interactions**	**Statistics**	
		***F***	***P***
BV/TV	BW*VWR*AGE	4.81	0.034
**Trabecular bone parameters**	**Significant interactions**	**Statistics**	
		***F***	***P***
BV/TV	BW*AGE	5.41	0.0246
BMD	BW*VWR*AGE	7.36	0.0097
Tb.Sp	AGE*BW	4.57	0.0382
Conn.D	AGE*BW	9.27	0.0039
**Bone mechanical properties**	**Significant interactions**	**Statistics**	
		***F***	***P***
Plastic work to failure	BW*VWR	4.73	0.0487
Post yield displacement	BW*VWR	5.93	0.03

Abbreviations: BW: body weight effect; VWR: voluntary wheel running effect; AGE: aging effect; BV/TV: trabecular bone volume fraction; BMD: Bone mineral density; Tb.Sp: trabecular separation; Conn.D: Connectivity density.

### The effects of long-term voluntary wheel running, aging, and body weight on profiles of muscle force-frequency and dependence on extracellular calcium

We further evaluated the intrinsic aspects of muscle excitation-contraction coupling by measuring relative muscle force production across increasing frequencies of stimulation (force-frequency). The force-frequency in the 18 mo old HBW group with VWR showed a slight rightward shift in soleus muscle relative force at the submaximal stimulatory frequencies of 40 Hz (CTRL: 49.9 ± 3.54 % and VWR 45.7 ± 7.26 %; HBW, *p* < 0.01, Data not shown) and 60 Hz (CTRL: 73.3 ± 2.54 % and VWR 69.8 ± 5.32 %; HBW, *p* < 0.05, Data not shown). EDL muscles did not differ in response to stimulation frequency with VWR at 18 mo or 22 mo of age.

Utilization of extracellular calcium is important to maintain muscle contractile function under repetitive activity or long-term activity via store-operated calcium entry (SOCE). Furthermore, SOCE is reduced in aged muscles [41, 42]. Muscles were thus subjected to calcium-depleted conditions followed by reintroduction of calcium to gauge the effects of VWR upon slow and fast-twitch muscle dependence and utilization of extracellular calcium. No changes were observed in muscle performance across the CBW groups, however when stratified by body weight, VWR in the 22 mo old LBW group improved soleus muscle force production during calcium-depleted conditions at maximal force (CTRL: 43.4 ± 5.44 % (of initial force) and VWR 59.8 ± 5.89 %; LBW, *p* < 0.0001, Data not shown) and submaximal force (CTRL: 28.6 ± 11.1 % (of initial force) and VWR 46.8 ± 3.54 %; LBW, *p* < 0.001, Data not shown). In contrast, HBW was associated with worse maximal force during calcium-depleted conditions in 22 mo old VWR soleus (CTRL: 46.8 ± 9.43 % (of initial force) and VWR 35.4 ± 4.08 %; HBW, *p* < 0.01, Data not shown) and EDL muscles (CTRL: 80.5 ± 25.8 % (of initial force) and VWR 43.1 ± 9.16 %; HBW, *p* < 0.01, Data not shown). Recovery of force following restoration of normal calcium concentration did not significantly differ in soleus and EDL muscles after VWR compared to CTRL (Data not shown). Multivariable regression analysis with interactions showed similar results as our initial analysis. A significant three-way interaction among body weight, VWR and age was detected in the soleus muscle (Table 2, *p* < 0.05) with LBW mice showing better force production during calcium-depletion and recovery after VWR compared to HBW mice (*p* = 0.0023–0.016). For EDL muscle, there was a two-way interaction between body weight and age for muscle response to calcium-depleted conditions and recovery period (Table 2, *p* < 0.05). These studies also indicate that the soleus muscles under all conditions have a greater dependence on SOCE than EDL muscles.

### Long-term voluntary wheel running during aging improves EDL muscle resistance to fatigue in 18 mo LBW mice and recovery from fatigue in HBW mice

Elderly individuals experience an increased risk of falls due to loss of muscle coordination and postural control. Acute muscle fatigue may play a significant role in this susceptibility to falls in the elderly and contribute to ability to stay active quality of life [43]. Therefore, we asked whether long-term endurance exercise with aging in mice could improve muscle fatigue resistance and the ability to recover following a bout of fatigue. Soleus muscles showed no significant impact of VWR on muscle fatigue profiles (Supplementary Figure 6A–6D, *p* > 0.05). Conversely, EDL muscle (Supplementary Figure 6E–6H) from 18 mo old VWR mice showed significantly improved resistance to acute fatigue as evidenced by 15% and 19% greater force in the CBW and LBW groups, respectively, after the initial 30 seconds of the fatiguing protocol, with no changes occurring with VWR in the HBW group. Improvements to EDL muscle fatigue resistance in the CBW and LBW groups were not apparent in the 22 mo animals that underwent 10 mo VWR. Instead, we found that VWR in 22 mo old HBW mice was associated with impaired fatigue resistance at the early phase of fatigue (Supplementary Figure 6H, HBW, *p* < 0.05). Our follow-up analysis using a multivariable regression model with interactions determined a two-way interaction among body weight and age for soleus muscle fatigue (Table 2, *p* < 0.05) showing an increase in fatigue resistance with aging from 18 mo to 22 mo in LBW mice (*p* = 0.0049). In EDL muscle, on the other hand, there were no significant interactions found for fatigue (*p* > 0.05).

VWR had different impacts on the recovery of muscle force after fatigue dependent upon muscle type, body weight grouping and age. As seen in Supplementary Figure 7, 18 mo old VWR mice had reduced soleus muscle recovery from fatigue at submaximal force in the LBW group only (LBW, *p* < 0.05), while 22 mo old VWR mice showed reduced recovery from fatigue at submaximal force in the HBW group only (HBW, *p* < 0.05). Next, EDL muscles from 18 mo old VWR mice in the HBW group showed enhanced recovery of force after fatigue at maximal and submaximal force when treated with the calcium release agent, caffeine (HBW*, p* < 0.05). On the other hand, VWR in 18 mo old LBW mice led to lower force recovery following caffeine treatment compared to CTRL (LBW, *p* < 0.05). Although body weight grouping showed differences with VWR in the ability of muscles to recover after fatigue, no significant interaction effects among body weight, VWR or age factors were detected using the multivariable regression approach (*p* > 0.05).

### Effects of 6 mo of VWR on skeletal muscle fiber type, cross-sectional area and number

**Figure 6:**
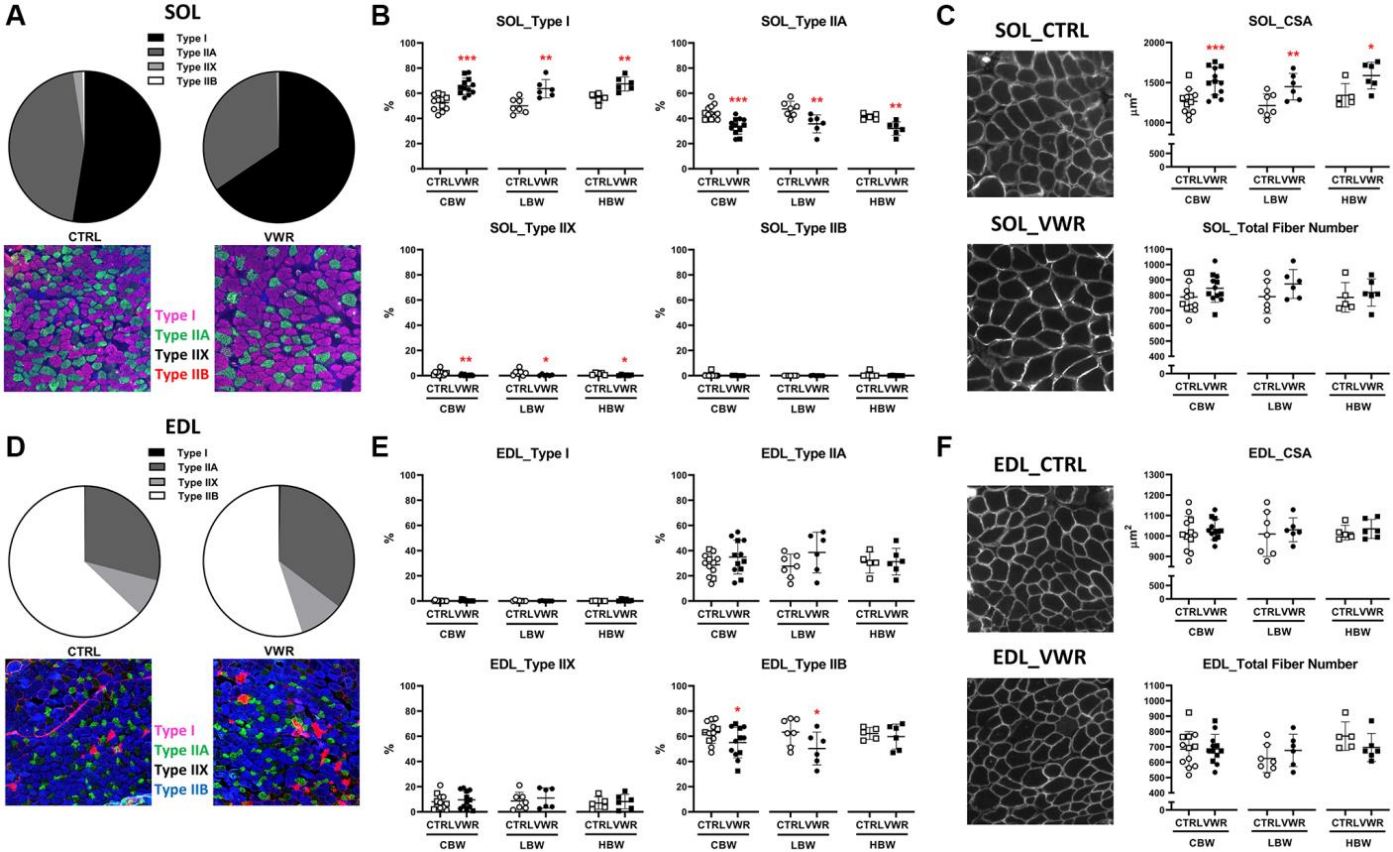
**Effects of endurance exercise on skeletal muscle fiber type, cross-sectional area and number in 18 mo old mice.** Endurance exercise induced fiber type switching from Type II to Type I in the soleus (SOL) but a reduction in Type IIB in the EDL in 18 mo old mice. Endurance exercise increased fiber cross-sectional area (CSA) in SOL, but not EDL in 18 mo old mice. No significant differences were found in the total fiber number of both skeletal muscles. (**A**, **B**) SOL fiber typing. (**A**) Pie charts showing all mice (CBW) in the CTRL and all in the VWR groups with the representative images. (**B**) The effect of VWR on each fiber type in different subgroups, CBW, LBW and HBW mice. ^*^: *p* < 0.05, ^**^: *p* < 0.01, and ^***^: *p* < 0.001, VWR vs. CTRL mice. (**C**) The effect of VWR on SOL fiber CSA and number with representative images. ^*^: *p* < 0.05, ^**^: *p* < 0.01, and ^***^: *p* < 0.001, VWR vs. CTRL mice. (**D**, **E**) EDL fiber typing. (**D**) Pie charts showing all mice (CBW) in the CTRL and in the VWR with the representative images. (**E**) The effect of VWR on each fiber type in two different subgroups, LBW and HBW mice. ^*^: *p* < 0.05, VWR vs. CTRL mice. (**F**) The effect of VWR on EDL fiber CSA and number with the representative images. No significant differences were found. Abbreviations: CTRL: control group; VWR: voluntary wheel running group; CBW: Combined groups (18 mo: *n* = 12 CTRL, *n* = 12 VWR); LBW: Low body weight group (18 mo: *n* = 7 CTRL, *n* = 6 VWR); HBW: High body weight group (18 mo: *n* = 5 CTRL, *n* = 6 VWR).

As endurance exercise is well known to induce fiber type switching and increase fiber cross-sectional area in muscle, these parameters were quantitated for both soleus (Figure 6A and 6B) and EDL (Figure 6D and 6E) from 18 mo old mice. The histological analysis shows that VWR was associated with a greater amount of slow-oxidative muscle fibers and less fast-glycolytic fibers compared to CTRL. In soleus muscle from VWR mice, there was a significant increase in the proportion of Type I fibers from 52.6% ± 5.8 to 65.6% ± 6.5 (CBW, *p* < 0.001), while Type IIA and Type IIX were decreased from 45.0% ± 5.7 to 34.0% ± 6.3 (CBW, *p* < 0.001) and from 2.0% ± 1.9 to 0.5% ± 0.5 (CBW, *p* < 0.01), respectively. No change was observed in Type IIB fibers. Similar fiber type switching was confirmed in both LBW or HBW subgroups (Figure 6B), showing no effect of body weight on fiber switching. A significant reduction in Type IIB was observed in the EDL CBW group with VWR (*p* < 0.05), which was reproduced in the LBW (*p* < 0.05) but not in the HBW subgroup (Figure 6E), suggesting a potential effect of body weight on EDL fiber switching. This was accompanied by a trend toward increasing numbers of type IIA fibers, which did not reach significance. Consistent with the effect of VWR to increase soleus muscle weight, increased muscle fiber cross-sectional area was observed in soleus muscles with VWR in CBW (*p* < 0.001) by around 20%, and in both LBW (*p* < 0.01) or HBW mice (*p* < 0.05) subgroups, with no significant change in the total fiber number (Figure 6C). Consistent with the lack of effect of VWR on EDL muscle weight, there was no significant change in fiber cross-sectional area or fiber number in EDL muscle with VWR in either the CBW, LBW or HBW groups (Figure 6F). In summary, long-term endurance exercise induced fiber type switching and increased fiber cross-sectional area in the soleus of 18 mo old mice.

### Maintenance of skeletal muscle-secreted osteocyte protective factors by long-term voluntary wheel running in aged LBW mice

**Figure 7:**
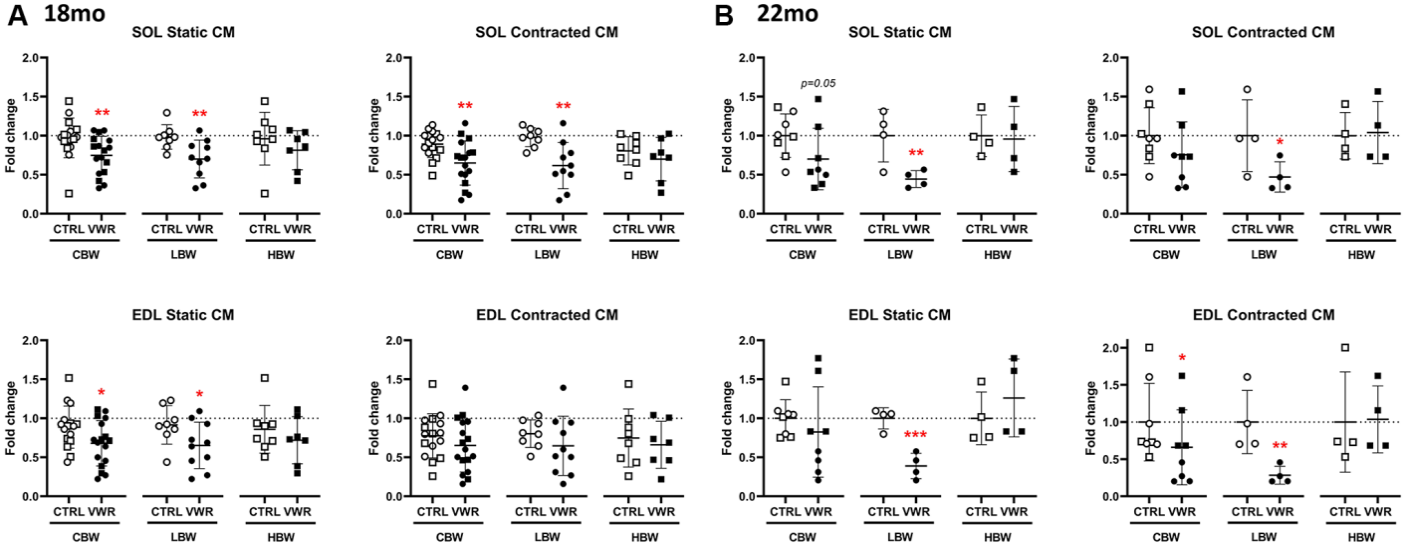
**Maintenance of skeletal muscle-secreted osteocyte protective factors by long-term voluntary wheel running in aged LBW mice.** Endurance exercise restored skeletal muscle-secreted osteocyte protective factors in aged 18 mo and 22 mo old LBW, but not HBW mice. (**A**, **B**) The effect of VWR on osteocyte protective factors in each skeletal muscle CM under *ex vivo* static and contracted conditions obtained from two different subgroups, LBW and HBW mice. (**A**) 18 mo and (**B**) 22 mo mice. ^*^: *p* < 0.05, and ^**^: *p* < 0.01, VWR vs. CTRL mice. Abbreviations: CTRL: control group; VWR: voluntary wheel running group; CBW: Combined groups (18 mo: *n* = 16 CTRL, *n* = 17 VWR; 22 mo: *n* = 8 CTRL, *n* = 8 VWR); LBW: Low body weight group (18 mo: *n* = 8 CTRL, *n* = 10 VWR; 22 mo: *n* = 4 CTRL, *n* = 4 VWR); HBW: High body weight group (18 mo: *n* = 8 CTRL, *n* = 7 VWR; 22 mo: *n* = 4 CTRL, *n* = 4 VWR).

Previously we showed that the skeletal muscle of young adult mice secretes osteocyte-protective factors that are increased with muscle contraction [18, 19] and that aging diminishes the protective effect of muscle-secreted factors against reactive oxygen species, ROS, induced osteocyte cell death [44]. We therefore sought to answer whether long-term physiological exercise could maintain the capacity of muscle to secrete factors that maintain osteocyte viability. Conditioned media (CM) from both static and contracted SOL skeletal muscle isolated from 18 mo VWR mice was significantly more protective against oxidative stress induced osteocyte cell death (around 25%) compared to the corresponding CTRL CM in the CBW group and this was maintained in the LBW subgroup but not the HBW subgroup (Figure 7A). In EDL, the static but not contracted CM from the VWR 18 mo animals significantly protected against osteocyte cell death in the CBW group and the LBW subgroup, but not the HBW subgroup (Figure 7A). These data suggest a beneficial effect of low body weight. In the 22 mo old cohort, VWR maintained the protective effect of muscle CM on osteocyte cell death in the LBW subgroup for both static and contracted SOL and EDL (Figure 7B). This suggests that obesity interferes with the production of muscle protective factors.

TUNEL staining of femurs to detect osteocyte apoptosis was performed to determine whether long-term physiological exercise prevents aging-induced osteocyte apoptosis *in vivo*. The state of the cells was classified into four categories, Category I: live, Category II: dying, Category III: apoptotic, and Category IV: empty lacunae. There were no differences in any category between CTRL and VWR groups (Supplementary Table 2). VWR showed a trend towards decreased apoptosis in the 18 mo HBW/VWR but this was not significant. Therefore, even though VWR maintained muscle produced factors that protect osteocytes from experimentally induced oxidative stress, this was not reflected by a significant reduction in osteocyte death in the femur *in vivo*.

### Long-term voluntary wheel running reduced aging-induced loss of dendrite numbers in the 22 mo HBW mice

**Figure 8:**
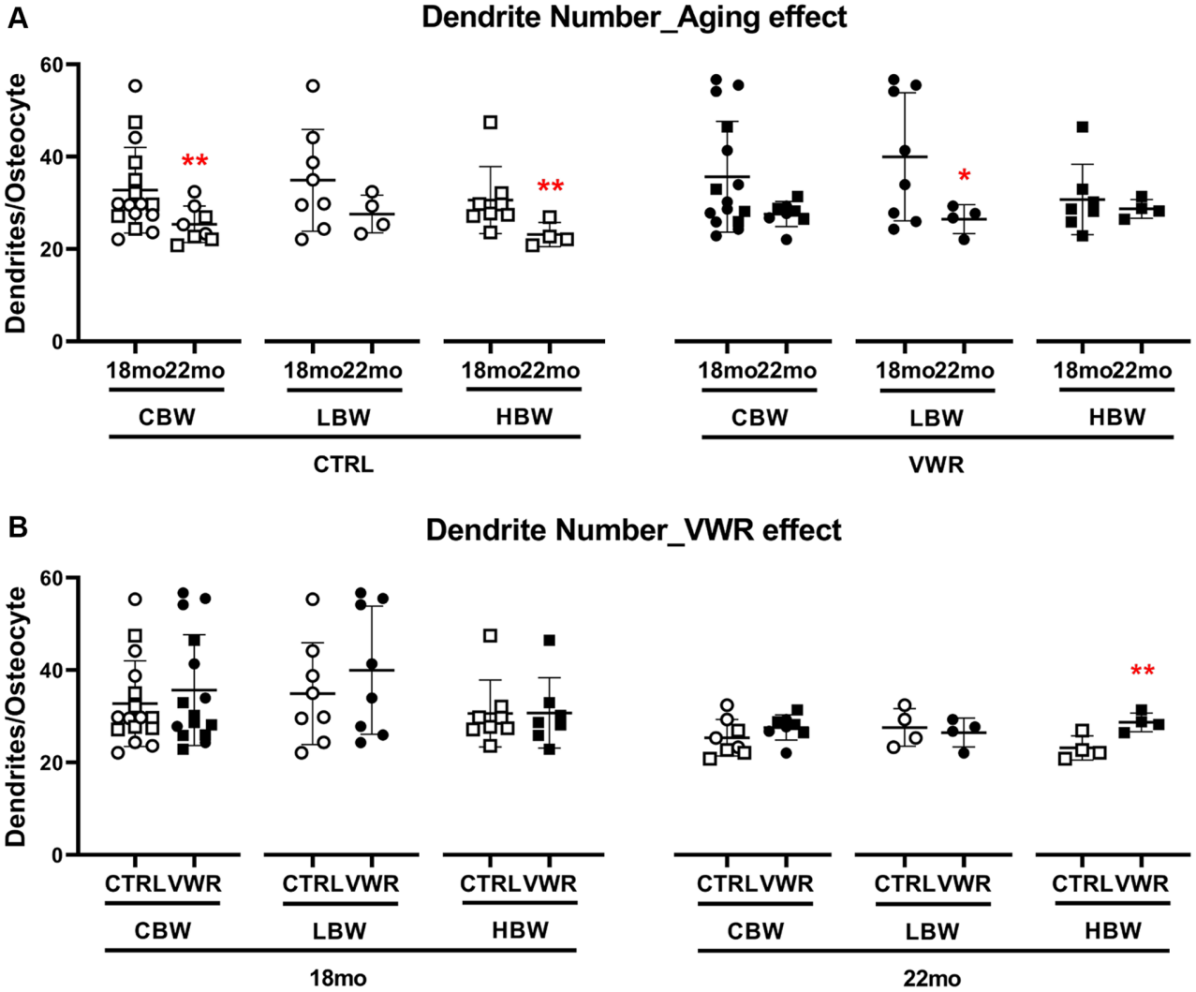
**Long-term voluntary wheel running reduced aging-induced loss of dendrite numbers in the 22 mo HBW mice.** 10 mo endurance exercise reduced advanced aging-induced loss of dendrite numbers in the 22 mo HBW/VWR. (**A**) The effect of advanced aging on osteocyte dendrite number in two different subgroups, LBW and HBW mice. ^*^: *p* < 0.05, 22 mo vs. 18 mo mice. (**B**) The effect of VWR on osteocyte dendrite number in two different subgroups, LBW and HBW mice. ^**^: *p* < 0.01, VWR vs. CTRL mice. Abbreviations: CTRL: control group; VWR: voluntary wheel running group; CBW: Combined groups (18 mo: *n* = 16 CTRL, *n* = 15 VWR; 22 mo: *n* = 8 CTRL, *n* = 8 VWR); LBW: Low body weight group (18 mo: *n* = 8 CTRL, *n* = 8 VWR; 22 mo: *n* = 4 CTRL, *n* = 4 VWR); HBW: High body weight group (18 mo: *n* = 8 CTRL, *n* = 7 VWR; 22 mo: *n* = 4 CTRL, *n* = 4 VWR).

We previously reported that osteocyte density and dendrite numbers were diminished during aging [45]. Therefore, we asked whether long-term physiological endurance exercise could prevent the aging-induced reduction in osteocyte dendrite numbers. Advanced aging significantly decreased osteocyte dendrite numbers by 23.2% in CTRL/CBW (18 mo: 33.0 ± 10.3 and 22 mo: 25.4 ± 4.0) and by 24.7% in CTRL/HBW mice (18 mo: 30.7 ± 7.8 and 22 mo: 23.2 ± 2.6, Figure 8A). This significant difference was lost with VWR in the HBW mice (18 mo: 32.6 ± 10.1 and 22 mo: 28.7 ± 2.0). VWR reduced dendrite loss in the 22 mo HBW subgroup (Figure 8B). These data suggest that VWR reduces dendrite loss in HBW mice. There were no differences in osteocyte density between LBW and HBW mice at either 18 mo or 22 mo and whether sedentary or exercising (data not shown).

### Long-term voluntary wheel running reduced aging-induced bone loss in 22 mo LBW but not HBW mice

The femoral cortical bone parameters (BV/TV: bone volume; BMD: bone mineral density; Co.Th: cortical thickness; Co.Area: cortical area; Pe.Cir: periosteal circumference; En.Cir: endosteal circumference) were measured by microCT and statistical analysis was performed to examine the effects of long-term voluntary wheel running. We observed a significant three-way interaction among body weight, VWR and age in cortical BV/TV (Table 2, *p* < 0.05).

#### Aging effects on femoral cortical parameters

**Figure 9:**
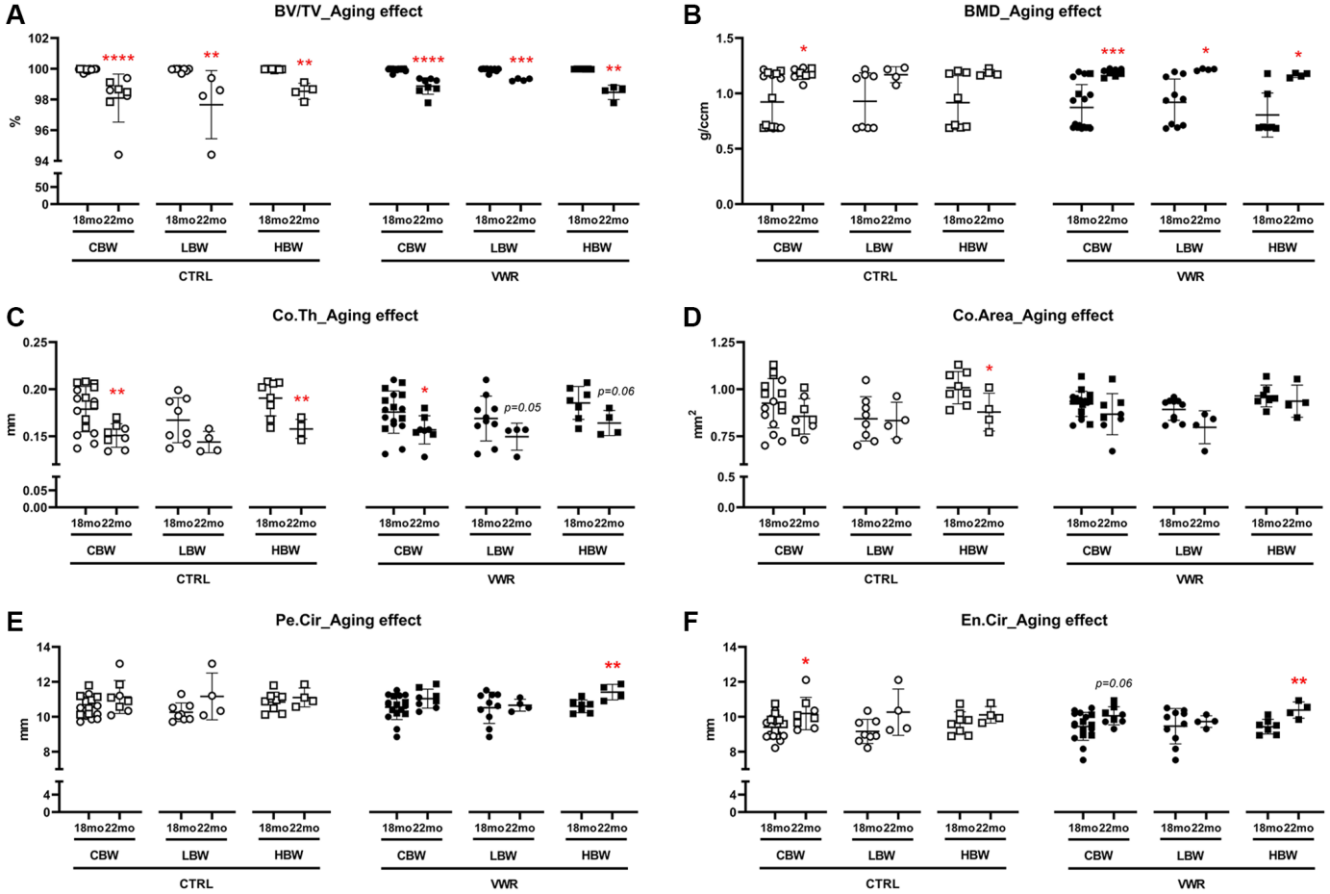
**Aging effects on femoral cortical parameters.** Aging effects were observed in BV/TV in LBW and HBW subgroups of 22 mo CTRL mice, Co.Th and Co.Area in 22 mo CTRL/HBW, BV/TV and BMD in LBW and HBW of 22 mo VWR mice, Pe.Cir and En.Cir in 22 mo VWR/HBW mice. Femoral cortical bone parameters, (**A**) bone volume fraction BV/TV, (**B**) bone mineral density BMD, (**C**) cortical thickness Co.Th, (**D**) cortical area Co.Area, (**E**) periosteal circumference Pe.Cir., and (**F**) endosteal circumference En.Cir, were measured by microCT. ^*^: *p* < 0.05, ^**^: *p* < 0.01, and ^***^: *p* < 0.001, 22 mo vs. 18 mo mice. Abbreviations: CTRL: control group; VWR: voluntary wheel running group; CBW: Combined groups (18 mo: *n* = 16 CTRL, *n* = 17 VWR; 22 mo: *n* = 8 CTRL, *n* = 8 VWR); LBW: Low body weight group (18 mo: *n* = 8 CTRL, *n* = 10 VWR; 22 mo: *n* = 4 CTRL, *n* = 4 VWR); HBW: High body weight group (18 mo: *n* = 8 CTRL, *n* = 7 VWR; 22 mo: *n* = 4 CTRL, *n* = 4 VWR).

We examined aging effects by comparing the 18 and 22 mo old animals (Figure 9). Advanced aging, 22 mo compared to 18 mo old mice, significantly reduced BV/TV in all CBW, LBW and HBW subgroups in CTRL and VWR mice. However, the differences were very small, indicating cortical bone porosity. Indeed, cortical porosity analysis revealed significant aging effects on porosity area in CBW and HBW subgroups in CTRL, and all CBW, LBW and HBW subgroups in VWR mice (Supplementary Figure 8A). However, the porosity surface was decreased except in HBW subgroups, suggesting that with aging smaller porosities enlarge and merge together resulting in reduced porosity surface in LBW (Supplementary Figure 8B). The aging-induced loss of BV/TV was reduced by VWR in LBW but not HBW of 22 mo VWR mice, suggesting that VWR prevents aging-increased bone porosities in only the LBW subgroup. (Figure 9A). In contrast, BMD was increased with aging regardless of VWR and body weight (Figure 9B). Co.Th was reduced in CBW of 22 mo CTRL mice than 18 mo (*p* < 0.01, Figure 9C), resulting from enlarged En.Cir (*p* < 0.05, Figure 9F). 22 mo CTRL/HBW clearly showed significant decreases in both Co.Th (*p* < 0.01) and Co.Area (*p* < 0.05) compared to 18 mo CTRL/HBW but no differences in the LBW subgroup (Figure 9C and 9D), accompanied by no changes in Pe.Cir (Figure 9E and 9F), indicating the beneficial effects of higher body weight on cortical thickness and area was lost during advanced aging due to the lack of periosteal expansion compensating for the loss. The aging-induced reduction in Co.Th and Co.Area was lost after 10 mo VWR in 22 mo VWR/HBW (Figure 9C and 9D). Moreover, the additional 4 mo VWR demonstrated a higher Pe.Cir in 22 mo HBW compared to 18 mo (*p* < 0.01, Figure 9E and 9F), suggesting VWR promoted periosteal expansion to improve cortical thickness and area in the HBW mice (Figure 9D and 9E).

#### Body weight effects on femoral cortical parameters

**Figure 10:**
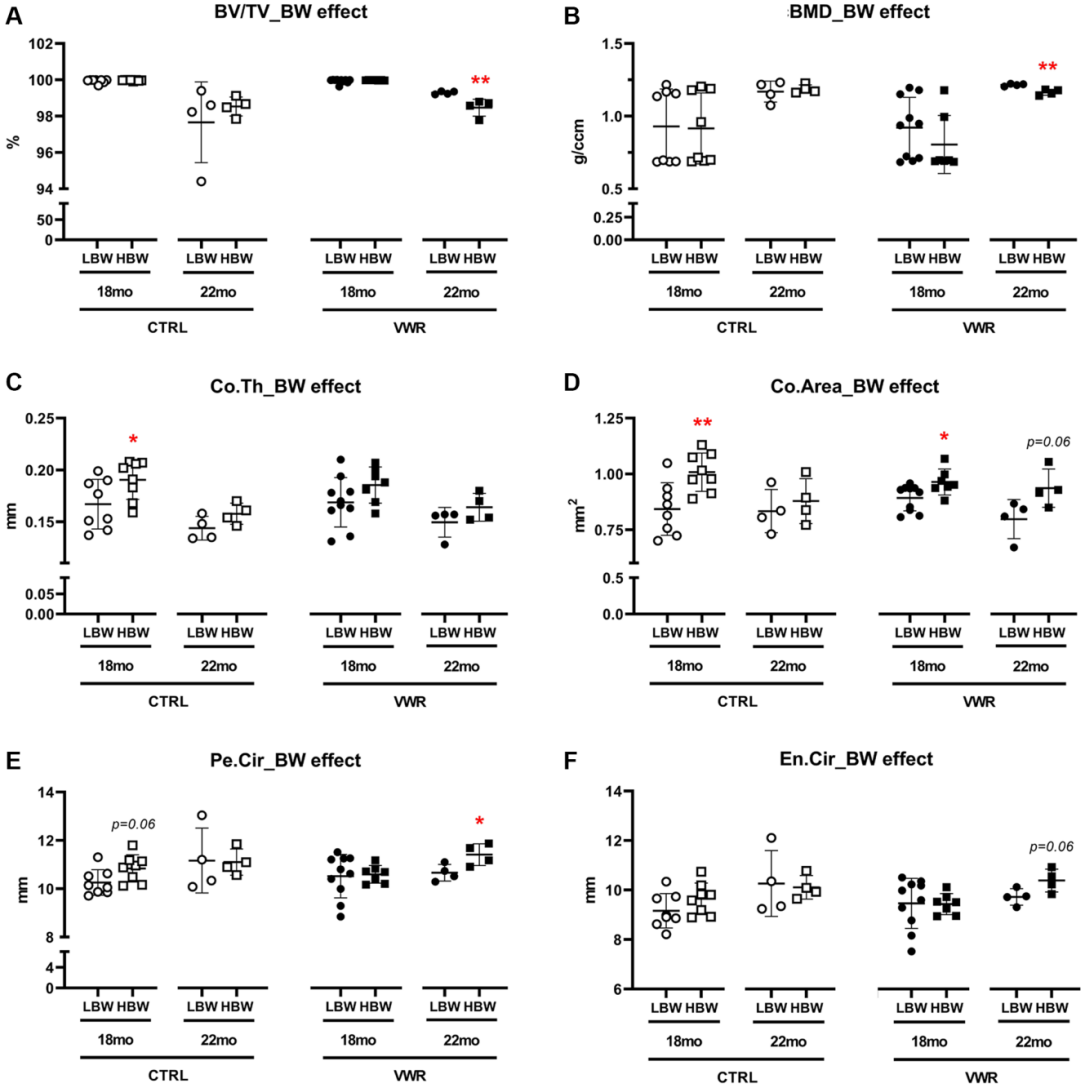
**Body weight effects on femoral cortical parameters.** The effect of body weight (BW) on femoral cortical parameters in two different age groups, 18 mo and 22 mo mice. BW effect was observed in Co.Th and Co.Area in 18 mo CTRL/HBW mice and BV/TV, BMD, Pe.Cir in 22 mo VWR/HBW mice. (**A**) bone volume fraction (BV/TV), (**B**) bone mineral density (BMD), (**C**) cortical thickness (Co.Th), (**D**) cortical area (Co.Area), (**E**) periosteal circumference (Pe.Cir), and (**F**) endosteal circumference (En.Cir), were measured by microCT. ^*^: *p* < 0.05, and ^**^: *p* < 0.01, HBW vs. LBW mice. Abbreviations: CTRL: control group; VWR: voluntary wheel running group; CBW: Combined groups; LBW: Low body weight group (18 mo: *n* = 8 CTRL, *n* = 10 VWR; 22 mo: *n* = 4 CTRL, *n* = 4 VWR); HBW: High body weight group (18 mo: *n* = 8 CTRL, *n* = 7 VWR; 22 mo: *n* = 4 CTRL, *n* = 4 VWR).

Significant effects of body weight were found in both Co.Th (*p* < 0.05) and Co.Area (*p* < 0.01) in 18 mo old CTRL mice, 14.1% and 19.6% higher in HBW than LBW subgroup, respectively (Figure 10C and 10D). This may be due to an increasing trend in Pe.Cir in 18 mo CTRL/HBW (Figure 10E). It suggests that higher body weight positively affects cortical thickness and area in sedentary conditions. However, after 6 mo VWR, the positive effects of higher body weight on Co.Th and Co.Area were lost or reduced in the HBW subgroup, indicating that higher body weight may have an adverse impact on cortical thickness and area during exercise. After 10 mo VWR, the HBW subgroup showed significantly lower BV/TV (*p* < 0.01) and BMD (*p* < 0.01) but higher Pe.Cir (*p* < 0.05) compared to the LBW subgroup (Figure 10A, 10B, and 10F). This suggests that an additional 4 mo VWR promoted further structural modifications to compensate for the increased bone porosities and lower BMD in HBW but not the LBW mice. We did not observe any significant differences in osteoclast number and surface on the femoral midshaft endocortical surface in any group (Supplementary Figure 9).

#### Trabecular bone parameters

Table 3 shows trabecular bone parameters of microCT analysis of femurs, and Table 2 for interaction analysis results. BMD showed a three-way interaction among body weight, VWR and age (*p* < 0.01). BMD was increased by 2.4-fold in 22 mo CTRL/CBW compared to 18 mo CTRL/CBW mice, primarily due to the HBW subgroup (3.5-fold), suggesting that higher body weight has a positive effect on trabecular bone mineral density. Two-way interactions between age and body weight were observed in BV/TV (*p* < 0.05), Tb.Sp (*p* < 0.05) and Conn.D (*p* < 0.01). A 6.6-fold higher Tb.N (*p* < 0.05) was observed in HBW compared to the LBW subgroup in 22 mo CTRL mice. Tb.Sp, was 24% lower in HBW than in the LBW subgroup in 22 mo VWR mice. These results indicate that higher body weight positively affects trabecular bone parameters. However, VWR did not significantly affect them compared to CTRL.

**Table 3:** μCT analysis of femurs from LBW/HBW Mice with or without long-term endurance exercise.

**GROUP**	**18 mo old**		**22 mo old**	
	**CTRL**	**VWR**	**CTRL**	**VWR**
CBW	*N* = 16	*N* = 17	*N* = 8	*N* = 8
LBW	*N* = 8	*N* = 10	*N* = 4	*N* = 4
HBW	*N* = 8	*N* = 7	*N* = 4	*N* = 4
**TRABECULAR BONE PARAMETERS**				
**BV/TV (%)**				
CBW	3.34 ± 3.10	1.65 ± 1.26	3.10 ± 3.82	1.46 ± 1.09
	(0.00–11.56)	(0.00–4.94)	(0.39–12.04)	(0.21–3.27)
LBW	4.30 ± 3.99	1.46 ± 1.37	0.94 ± 0.66	0.96 ± 0.34
	(0.14–11.56)	(0.00–0.62)	(0.39–1.87)	(0.73–1.46)
HBW	2.38 ± 1.60	1.92 ± 1.14	5.25 ± 4.60	1.96 ± 1.42
	(0.00–4.29)	(0.57–4.03)	(1.82–12.04)	(0.21–3.27)
**Tb.Th (mm)**				
CBW	0.05 ± 0.02	0.05 ± 0.02	0.05 ± 0.01	0.06 ± 0.01
	(0.00–0.07)	(0.00–0.08)	(0.03–0.07)	(0.04–0.07)
LBW	0.05 ± 0.01	0.05 ± 0.02	0.05 ± 0.02	0.06 ± 0.01
	(0.02–0.06)	(0.00–0.08)	(0.03–0.07)	(0.05–0.07)
HBW	0.05 ± 0.02	0.05 ± 0.01	0.05 ± 0.01	0.05 ± 0.01
	(0.00–0.07)	(0.04–0.06)	(0.04–0.06)	(0.04–0.06)
**Tb.N (1/mm)**				
CBW	1.28 ± 1.49	0.80 ± 0.73	0.65 ± 0.98	0.26 ± 0.19
	(0.00–5.75)	(0.00–1.93)	(0.11–3.03)	(0.06–0.59)
LBW	1.72 ± 1.92	0.56 ± 0.66	0.17 ± 0.07	0.16 ± 0.04
	(0.06–5.75)	(0.00–1.69)	(0.11–0.26)	(0.12–0.21)
HBW	0.84 ± 0.78	1.14 ± 0.72	1.13 ± 1.27 ^b^	0.34 ± 0.23
	(0.00–1.95)	(0.11–1.93)	(0.33–3.03)	(0.06–0.59)
**Tb.Sp (mm)**				
CBW	0.45 ± 0.20	0.56 ± 0.20^a^	0.52 ± 0.21	0.63 ± 0.12
	(0.00–0.76)	(0.00–0.77)	(0.19–0.75)	(0.47–0.80)
LBW	0.43 ± 0.14	0.51 ± 0.22	0.58 ± 0.22	0.71 ± 0.08
	(0.18–0.65)	(0.00–0.74)	(0.28–0.75)	(0.63–0.80)
HBW	0.48 ± 0.25	0.63 ± 0.14	0.45 ± 0.20	0.54 ± 0.08^b^
	(0.00–0.76)	(0.38–0.77)	(0.19–0.66)	(0.47–0.63)
**BMD (g/ccm)**				
CBW	0.05 ± 0.03	0.04 ± 0.02	0.12 ± 0.05^c^	0.11 ± 0.03^c^
	(0.00–0.09)	(0.00–0.08)	(0.07–0.20)	(0.07–0.16)
LBW	0.06 ± 0.02	0.04 ± 0.03	0.11 ± 0.44	0.13 ± 0.03^c^
	(0.04–0.09)	(0.00–0.08)	(0.07–0.15)	(0.09–0.16)
HBW	0.04 ± 0.02	0.04 ± 0.01	0.14 ± 0.05^c^	0.09 ± 0.02^c^
	(0.00–0.09)	(0.03–0.06)	(0.09–0.20)	(0.07–0.11)

Data are mean ± SD (range). Abbreviations: CTRL: control group; VWR: voluntary wheel running group; CBW: Combined groups; LBW: Low body weight group; HBW: High body weight group; BV/TV: trabecular bone volume fraction; Tb.Th: trabecular thickness; Tb.N: trabecular number; Tb.Sp: trabecular separation; BMD: Bone mineral density. ^a^*p* < 0.05 vs. Corresponding CTRL, ^b^*p* < 0.05 vs. Corresponding LBW, ^c^*p* < 0.05 vs. Corresponding 18 mo.

### Long-term voluntary wheel running altered tibial mechanical properties in 18 mo LBW but not HBW mice

**Figure 11:**
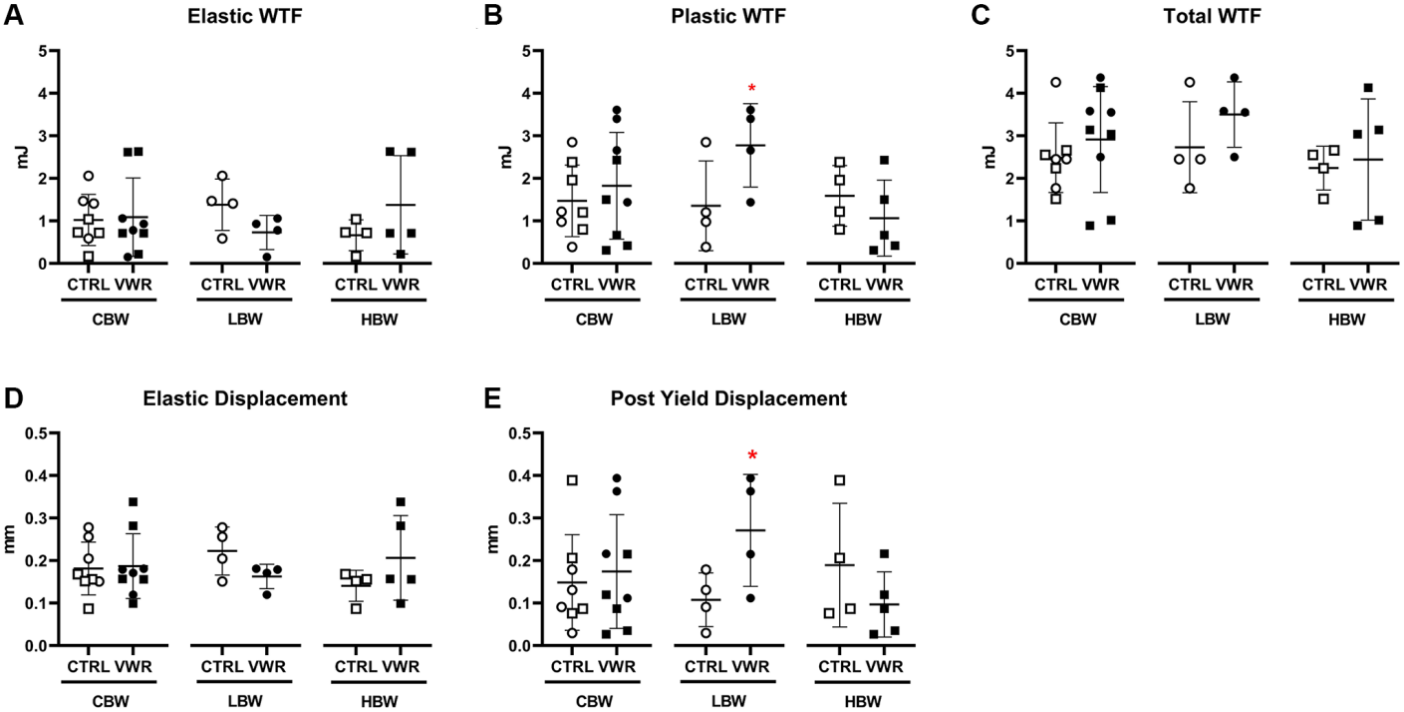
**Long-term voluntary wheel running altered femoral mechanical properties in 18 mo LBW, but not HBW mice.** 6 mo endurance exercise altered femur mechanical property to be more ductile in 18 mo LBW/VWR, but not HBW/VWR mice. (**A**) Elastic WTF, (**B**) Plastic WTC, (**C**) Total WTF, (**D**) Elastic displacement, (**E**) Post yield displacement. ^*^: *p* < 0.05, VWR vs. CTRL mice. Abbreviations: CTRL: control group; VWR: voluntary wheel running group; CBW: Combined groups (*n* = 8 CTRL, *n* = 9 VWR); LBW: Low body weight group (*n* = 4 CTRL, *n* = 4 VWR); HBW: High body weight group (18 mo: *n* = 4 CTRL, *n* = 5 VWR); WTF: work to failure.

To determine whether VWR had beneficial effects on bone strength, mechanical testing was performed on tibias from the 18 mo VWR animals. 6 mo VWR significantly increased both plastic work to failure (WTF) (2.1-fold; *p* < 0.05) and post yield displacement (2.5-fold; *p* < 0.05) in 18 mo LBW, but not 18 mo HBW mice (Figure 11B and 11E). No significant differences in elastic WTF, total WTF and elastic displacement were observed in either subgroup (Figure 11A, 11C and 11D). Other mechanical properties such as ultimate load, elastic stiffness, elastic modulus, and the moment of inertia were not significantly changed in either LBW or HBW mice (Supplementary Table 3). No significant differences were found in hardness using nanoindentation of femurs, but the trends were the opposite between the 18 and 22 mo old animals, with an upward trend in the 18 mo old and a downward trend in the 22 mo old animals (Supplementary Table 4), which is consistent with the decreased cortical BV/TV during the advanced aging from 18 mo to 22 mo.

## DISCUSSION

In this study, we examined the efficacy of a long-term VWR exercise program without the use of resistance initiated at mid-life on maintaining slow and fast-twitch skeletal muscle mass and contractility, muscle factor production, and bone quality into old age in female mice. We hypothesized that voluntary exercise would improve muscle function and that muscle-derived soluble factors produced with exercise would help to maintain bone cell viability and improve bone parameters. During our study, the animals displayed a wide variability in their final body weights and thus were divided into low body weight (LBW) and high body weight (HBW) subgroups based on the median of the final body weights for each group in order to account for the influence of body weight on skeletal muscle function and bone mass/BMD. Our results demonstrate differential interactions of body weight and age on skeletal muscle function, muscle factor release and bone properties after long-term VWR exercise in aging female mice.

### Influence of relative body weight and age on VWR effects

Body weight significantly modified the effects of VWR on a number of parameters without significantly affecting the distance run by the animals. For example, VWR for 6 mo until 18 mo increased heart weight/body weight ratio and decreased bone marrow fat only in the HBW but not LBW subgroups, suggesting that body weight influences the effects of running. Age and duration of exercise across life also influence the effects of VWR. VWR for 10 mo until 22 mo of age but not 6 mo until 18 mo significantly decreased body weight in both LBW and HBW subgroups. The most consistent and greatest effect of VWR was observed on muscle.

### Changes in muscle

Aging is accompanied by the progressive decline of skeletal muscle mass and contractile force or muscle strength which, in humans, typically begins after the fourth decade of life [46]. Maintaining adequate muscle function and physical capacity is an essential component of a healthy aging lifestyle which focuses on improving an individual’s well-being during the aging process. In this regard, we were interested in long-term voluntary endurance exercise beginning during middle-age, before skeletal muscle function begins to decline, and the effect of exercise on maintaining slow and fast-twitch muscle mass and contractile performance into old age. To our knowledge there are no other studies that have characterized muscle mass and contractile function of representative slow and fast-twitch muscles from mice performing long-term voluntary endurance exercise into old age together with the influence of body weight on these parameters. The findings from this study show beneficial effects of long-term voluntary exercise on increasing slow-twitch soleus muscle mass and contractile force production and improving resistance to fatigue in fast-twitch EDL muscle. The changes to muscle performance and size were associated with changes at the myofiber level including larger fiber cross-sectional area in the soleus and a more slow-oxidative fiber type profile in soleus and EDL muscles. Lastly, we show the impact of final body weight on the adaptation of slow and fast-twitch muscles to long-term VWR and its implications for aging and sarcopenia.

The ability to build muscle mass and strength is a primary outcome of concern when implementing exercise strategies in elderly individuals in order to mitigate the detrimental effects of age-associated muscle loss. Most studies focus on the utilization of resistance during exercise training in the sarcopenic elderly, however this option may not be entirely feasible in an elderly care community-dwelling setting [47]. Despite the fact that we did not include added resistance in our VWR protocol, our results show gains in slow-twitch soleus muscle mass that are in line with previous studies that have employed the use of resistance during long-term voluntary wheel exercise programs in rodents to induce muscle hypertrophy [48–50]. Having higher body weight was also found to have positive consequences for building soleus muscle mass and strength with VWR exercise during aging than having low body weight as evidenced by the statistically significant gains in these parameters in HBW mice from 18 mo to 22 mo but not in the LBW mice. Thus, it may be important to consider body weight or other measures of body size such as BMI as factors in the selection of exercise regimens for the elderly in order to help ensure the desired muscle-related outcomes.

Although we detected less direct changes from VWR in the fast-twitch EDL muscle mass with our non-resistance VWR protocol compared to slow-twitch soleus muscle, there were important enhancements in parameters of EDL size and contractile function with VWR during aging which were also influenced by body weight. Improved EDL fatigue resistance was observed in 18 mo old VWR mice in the LBW group, which may confer an advantage in helping to counteract the aging-associated fatiguability. Next, VWR abrogated the aging-related declines of fast-twitch EDL muscle mass and contractile strength that occurred from 18 mo to 22 of age in sedentary mice with HBW mice showing protection against these age-related changes even without long-term VWR. These findings, combined with the data in soleus muscle, suggest that greater body weight, possibly through higher loading on muscle experienced during both sedentary activity and with voluntary running exercise, positively contributes to slow and fast-twitch muscle mass and force output during the aging process [51, 52]. Interestingly, elevated body mass has also been associated with protective effects on outcomes of individuals with cardiovascular disease [53]. While HBW may provide an advantage for helping to increase and maintain muscle mass and strength during the aging process, other functional properties such as response to calcium depletion, fatigue resistance and recovery from fatigue were negatively affected by HBW at 22 mo of age in response to VWR in our study. Further research will be needed to determine how body-weight dependent differences in the adaptation of slow-and fast twitch muscle observed in this study translate to functional aspects of healthy aging and prevention of sarcopenia in human populations.

Falls are a leading cause of injury and mortality in older persons worldwide. Fatigue is considered a significant risk factor for falls in the elderly and is the focus of therapeutic intervention strategies to maintain a quality active lifestyle and for preventing the mortality and morbidity associated with falls [43, 54, 55]. Muscular fatigue is characterized by an acute loss in muscle performance during repetitive activity and contributes to a reduction in postural control and balance. In our study, 18 mo old VWR mice had better fatigue resistance during the early phase of fatigue in the EDL muscle in the CBW group. When separated by body weight, the improvement to fatigue resistance with VWR was found in the LBW but not HBW mice. In addition, EDL muscles from 22 mo old HBW mice with VWR exhibited significantly lower fatigue resistance during the early phases of fatigue compared to sedentary HBW mice. Thus, our findings suggest that higher body weight may have a negative effect on the ability of fast-twitch skeletal muscle to adapt appropriately to aerobic exercise during old age. A study investigating long-term wheel running from 9 mo to 24 mo of age in male mice found improved EDL muscle fatigue resistance which was mediated by enhanced utilization of extracellular calcium [56]. In our muscle contractility experiments we also measured muscle force after external calcium-depleted conditions and after reintroduction of normal calcium levels, however we did not detect any differences in EDL muscle performance among 18 mo old VWR and sedentary mice that would indicate a correlation with the fatigue data. It is possible that the specific fatiguing stimulation protocol or experimental conditions may account for some of these differences.

Both soleus and EDL muscles showed changes to muscle fiber type composition with VWR. In 18 mo old mice, VWR was associated with a higher proportion of type I fibers and a reduction in type IIa and type IIx fibers in the soleus muscle. In the EDL muscle there was a lower proportion of type IIb fibers with VWR. Type I fibers are characterized by expression of slow-twitch MyHC type I and exhibit abundant mitochondria and oxidative metabolism, while type II fibers express the fast-twitch MyHC type II isoforms with a higher reliance on glycolytic metabolism [57, 58]. Therefore, the changes seen in soleus and EDL muscle fiber type proportion with VWR in our study reflect a less glycolytic and more oxidative fiber phenotype which would be expected. This is in line with other studies investigating lifelong voluntary wheel exercise in mice that have demonstrated a shift in muscle fiber metabolic profiles towards a more oxidative state and increased oxidative enzyme content [48, 59]. Based on these findings, the fiber type changes in EDL muscle from 18 mo old VWR mice may underlie the enhancements observed in EDL fatigue resistance. Moreover, the improvement in fatigue resistance in LBW but not HBW 18 mo old VWR mice parallel with a significantly reduced proportion of type IIb fibers and a trend for higher type IIa fiber content in LBW but not HBW mice. Although soleus muscles from 18 mo old VWR mice showed a greater proportion of type I fibers and less type IIa and type IIx fibers compared to controls, there were no alterations to muscle fatiguability or recovery following fatigue. Other studies examining soleus muscle function after exercise training have also found no improvement to fatigue properties [60–63]. It is possible that the concomitant increase in type I and decrease in type IIa muscle fibers led to zero net gain in fatigue resistance, since both muscle type fibers are fatigue resistant [64, 65]. Yet, another possibility, in the soleus muscles of exercised mice, there is likely a larger ATP requirement due to the larger muscle fiber cross-sectional area and contractile force output, which would require a more oxidative fiber type profile to maintain a similar relative decrease in contractile force during fatigue as that of non-exercised muscles [60].

### Effects of muscle factors on osteocytes

The major effect of VWR on the generation of muscle factors protective of osteocyte viability was in the LBW and not the HBW subgroups. Conditioned media from both static and contracted soleus skeletal muscle from both 6 and 12 mo of VWR had higher protective activity than that from sedentary controls. Generally, the same results were observed with the EDL but only with the LBW and not the HBW subgroup. This beneficial effect of low body weight on muscle factors suggests that obesity may interfere with the production of muscle protective factors. Indeed, obesity has previously been shown to be associated with altered levels of muscle factors in both humans [66] and rodent models. Studies in high fat diet-induced obese rats have demonstrated decreased expression of muscle factors including that of interleukin (IL)-6 and an impairment in the induction of muscle factor expression after exercise training [67, 68].

Muscle has been shown to produce factors that can have negative effects on bone such as myostatin but with exercise, positive effects are noted such as that seen with irisin [69]. We previously reported l-β-aminoisobutyric acid (l-BAIBA) is one of the factors released from contracted soleus and EDL to protect osteocytes from oxidative stress and that both soleus and EDL muscle released similar amounts of l-BAIBA during *ex vivo* contraction [18]. We also demonstrated that irisin, which is increased by exercise and muscle contraction [70] and considered as a biomarker of skeletal muscle performance in the elderly [71], has a positive effect on osteocyte viability [72]. In the present study, the maintenance of muscle protective factors with VWR seems to be independent of fiber type switching, hypertrophy, or strength.

Though VWR maintained muscle secreted protective factors against osteocyte cell death *in vitro*, no significant effects were observed on osteocyte viability *in vivo*. There was a trend (*p* < 0.06) for fewer apoptotic osteocytes in 18 mo HBW with VWR. Our *in vitro* cell death assays employed the use of a robust stimulus for apoptosis (H_2_O_2_), however aging represents a more prolonged process where the apoptotic stimuli may be more subtle over time. In addition, we have previously shown that aging blunts the responsiveness of osteocytes to muscle protective factors, such as _L_-BAIBA through downregulation of the expression of its receptor MRGPRD (Mas-related G-protein coupled receptor, type D) [18]. Therefore, it is possible that the bone in our aged animals had a diminished sensitivity to muscle-derived protective factors produced during exercise *in vivo*. It also may be possible that the production of muscle factors and their downstream impacts on health may differ when considering different exercise types (i.e., aerobic, resistance, or a combination thereof). Though no significant effect was observed on osteocyte viability *in vivo*, dendrite number was maintained with 10 mo of VWR in HBW mice. We previously demonstrated a decrease in osteocyte dendrite number with age in mice [45, 73] and a diminished lacunar-canalicular network had been shown in aged women, largely due to a decline in the canalicular areal fraction [74]. A mouse model of type-2 diabetes mellitus induced by high-fat diet profoundly affects the osteocyte network topology [75]. It is plausible that not only aging but also obesity/excess body fat may play roles in modifications of the osteocyte dendrite network.

### Changes in bone

In the present study, fewer effects were observed on trabecular bone as compared to cortical bone, most likely because the most trabecular bone is lost in the long bones of females by this age [76]. However, a clear aging effect was observed in trabecular bone mineral content, as reported previously [77]. The 22 mo mice had greater bone mineral density in the HBW subgroup under sedentary conditions, however the HBW subgroup responded to VWR with a decrease in bone mineral density in contrast to the LBW subgroup. Body weight had an influence on trabecular bone parameters, mainly in the 22 mo old mice, consistent with correlation analysis results. In sedentary conditions, HBW mice have a higher trabecular number than LBW mice, and under 10 mo VWR conditions, the HBW mice had a reduction in trabecular spacing compared to the LBW mice. This suggests that higher body weight protects against trabecular bone loss under both sedentary and wheel running conditions.

With regards to cortical bone, the effects of age were as expected, showing a significant decrease in cortical bone volume reflecting increased cortical bone porosities, cortical thickness and area, and an increase in bone mineral density and endosteal circumference between the 18 and 22 mo old animals. A decrease in cortical thickness and area of long bones has previously been reported during aging [78–83]. Compared to young adult mice, aged mice show smaller cortical area site-specifically. Cortical thickness in aged mice is less than that of the young adult mice, but the difference is uniform along the length [84]. Cortical thickness in females, which peaks at 6 mo of age, declines slightly in middle age but drastically during advanced aging [76]. Additionally, the current study revealed that the alterations were mainly observed in the HBW animals and may be resulted from the lack of periosteal expansion during advanced aging.

There was an effect of body weight alone in the 18 mo HBW mice with greater cortical thickness and area than the LBW under sedentary conditions. Higher body weight is known to affect bone microarchitecture by increasing cortical thickness and area [85–88]. This beneficial effect of higher body weight was lost during the advanced aging from 18 mo to 22 mo without exercise. However, VWR corrected the lower cortical thickness and area observed in 22 mo HBW sedentary animals by promoting structural modifications with periosteal expansion, which may compensate for the increased cortical bone porosities and lower BMD. VWR also reduced aging-induced loss of osteocyte dendrite numbers in 22 mo HBW mice. In contrast, VWR reduced aging-induced cortical BV/TV loss and maintained higher BMD in 22 mo LBW animals. VWR also increased plastic work to failure and post-yield displacement in 18 mo LBW but not HBW, suggesting that exercise may allow an increase in ductility in animals with lower body weight. Short-term exercises such as running and swimming have demonstrated a positive impact on post-yield toughness during growth and in young adult mice [89–93]. Consistently, high-fat diet-fed mice were previously reported to have lower type I collagen and post-yield displacement [94–96]. This shows an effect of HBW on mechanical properties.

Collectively, VWR has beneficial effects on bone health during advanced aging regardless of body weight, but VWR differentially alters bone parameters depending on body weight, with modifications in mechanical properties in LBW but structural modifications in HBW contributing to the prevention of osteopenia.

### Experimental limitations

One limitation of this study was the variability in body weights of mice within experimental groups at later stages of the experiment, even though the average body weights of the control and VWR groups were similar at the start of the experiment. The large variability in the final body weights in the control group led us to stratify the mice into two subgroups, LBW and HBW, according to the median of the final body weight. In future studies, recording of food intake and body composition could be performed over time, which would provide additional information for interpretation of the results.

We chose voluntary wheel running as a means of endurance exercise instead of treadmill running, swimming, wire-walking etc. based on the fact that voluntary running behavior is natural and not stressful for the animal and the running takes place during their normal hours of activity during the night, which is less disruptive to circadian rhythms, etc. This type of exercise is also more easily applied to long-term exercise studies due to less intervention by researchers [97]. However, a disadvantage of this approach is the lack of control over the duration of running. Our data showed a gradual decrease in running activity after the peak run distance of 6–9 km/day reached over the first 2–6 weeks of exercise, which slowed to around 2 km/day by the last month. There is likely a natural decline in desire and/or ability for aerobic exercise with aging. Although 2 km/day would still seem to be a significant amount of exercise in an aging mouse, under the current experimental design, the amount of running/cage activity of the sedentary controls is not known. If the run distance in the exercised mice could be maintained to a similar level to the peak during the entire experimental period, more beneficial effects of endurance exercise might have occurred on both bone and muscle. Additionally, the daily/weekly run distances revealed considerable variation between mice and between different experimental cohorts and even in the same mouse.

The influence of other systems, such as the nervous system, in the body is hard to determine. Our results in isolated skeletal muscles demonstrated beneficial alterations to intrinsic muscle contractile function in response to VWR during aging. However, in our model we were not able to account for changes in the motor neurons or at the neuromuscular junction which would affect muscle functional output. Along with the declines in muscle tissue and performance, aging is associated with a reduction in the number of functional motor neurons, their firing rate, and the degree of innervation of muscle fibers [98]. These neuromuscular changes in the elderly also contribute to greater muscle fatigue and reduced postural control and balance, which exacerbates risk of falling [99]. VWR exercise has been previously found to delay the losses to neuromuscular components during aging [100]. Thus, it is likely that in our study, VWR with aging produced neuromuscular adaptations that would further modulate muscle contractility *in vivo*. Nevertheless, our study focused specifically on bone and skeletal muscle.

Aging diminishes the skeleton’s adaptive response to mechanical loading to induce periosteal bone formation [101, 102]. Recent studies shed light on controlling bone loss in the elderly [103]. High intensity resistance and impact training improved BMD, cortical thickness and physical performance in postmenopausal women with low bone mass [104]. It is also reported that 20 wk high-intensity strength and sprint training in middle-aged and old male athletes demonstrated significant improvements in the mid-tibial structure and strength [105]. A brief high-magnitude, load-priming regime was shown to effectively induce a mechanoadaptive response in cortical bone in aged mice, which was associated with the downregulation of sclerostin in osteocytes [106]. Therefore, resistance exercises that induce higher strain magnitudes may be necessary to induce meaningful adaptation in bones.

Another limitation is the increased variability that occurs with aging studies. Like humans, as animals age, they become susceptible to disease and other environmental factors that can modify outcomes and increase the variability of measured endpoints. In spite of these limitations, the present study showed beneficial effects of VWR on both muscle and bone function. In summary, both long-term VWR and HBW were effective in maintaining soleus and EDL muscle mass and contractile force with age. In bone, HBW appears to have more positive effects under sedentary conditions and VWR has more positive effects in animals with LBW.

## MATERIALS AND METHODS

### Animals

Ten to eleven month old C57Bl6 female mice were obtained from the NIH/NIA aging colony via Charles Rivers Laboratories. All of the mice were given access to a voluntary wheel running (VWR) apparatus (Mouse Home Cage Running Wheel, Columbus Instruments, Columbus, OH, USA) in their cages for 1–2 wk, to determine which mice had a tendency to run. Each wheel has a magnetic indicator and a Hall effect sensor that connects to a computer interface and records wheel revolutions using the Multi-Device Interface MDI software (Columbus Instruments). The data was exported into a CSV format at the end of the experiment.

At approximately 12 mo of age, the mice that showed inclination to run were placed in the voluntary wheel running exercise group (VWR) and age and body weight-matched mice were used as controls. The Control group, “CTRL” was placed in standard mouse cages and housed under normal conditions, while the “VWR” group were placed in standard mouse cages containing the voluntary wheel running apparatus. These mice had unrestricted access to the wheel for 6 or 10 mo and were maintained on a 12 hr light/dark cycle at 22°C constant temperature and 45–55% humidity with standard lab chow *ad libitum*, before sacrificing at 18 or 22 mo of age, respectively. CTRL and VWR mice were individually housed throughout the experiment, monitored daily and weighed monthly, and were provided food and water *ad libitum*, in accordance to an approved IACUC protocol (UMKC IACUC Protocol 1301). On the day of sacrifice, final weights and a health report were recorded. Four independent experiments were performed for the 6 mo VWR and two for the 10 mo VWR, for a final sample size of 16–17 mice per group for 6 mo VWR and 8 mice per group for 10 mo VWR. The experiments were performed Oct. 2015 to April 2016, Aug 2016 to Feb 2017, July 2017 to Jan 2018 and Feb 2019 to July 2019. The data from the four running experiments in 18 mo old mice were averaged and data from the two running experiments in 22 mo old mice were averaged for analysis and presentation in this manuscript.

### *Ex vivo* skeletal muscle contractility

18 mo and 22 mo old VWR and CTRL mice were sacrificed by cervical dislocation and the soleus and extensor digitorum longus (EDL) muscles were removed for contractility analysis as previously described [18, 107–109]. Following mounting of muscles to the contractility system (GlobalTown Microtech, Sarasota, FL, USA) and preparation of muscle conditioned media (CM) as described below, contractile analysis was performed in physiological buffer (144 mM NaCl; 5 mM KCl; 1 mM MgCl2; 25 mM NaHCO3; 2.5 mM CaCl2; 10 mM glucose; pH 7.45) maintained at 37°C and aerated with 95%/5% O_2_/CO_2_. Soleus and EDL muscles were first allowed a 30 min equilibration period during which time they were contracted with maximal and submaximal-frequency stimulations with a three-minute rest interval (160/40 Hz for soleus; 200/100 Hz for EDL). Following equilibration, soleus and EDL muscles were stimulated to contract with frequencies ranging from 1–220 Hz to generate the force-frequency relationship. Next, physiological buffer was replaced with a calcium-depleted physiological buffer (144 mM NaCl; 5 mM KCl; 1 mM MgCl2; 25 mM NaHCO3; 0 mM CaCl2; 0.1 mM EGTA; 10 mM glucose; pH 7.45) and soleus and EDL muscles were contracted with alternating maximal and submaximal-frequency stimulations with a three-minute rest interval for 30 min. The buffer was then replaced with physiological buffer with normal calcium concentration and muscles were allowed to recover under continued maximal and submaximal contractions for 30 min. To induce fatigue, the muscles were next contracted with alternating maximal and submaximal stimulations for 5 min with a rest interval of two seconds. Immediately following the fatiguing protocol, muscles were allowed a 30 min recovery period while contracting at maximal and submaximal force with a rest period of 3 min, followed by 5 mM caffeine addition to the muscle contractility chambers to evaluate calcium availability during the recovery period. At the end of the experiment, soleus and EDL muscle optimal length and muscle weight were measured and left limb soleus and EDL muscles were snap frozen in liquid nitrogen and stored at −80°C while right limb soleus and EDL muscles were frozen in Tissue-Tek O.C.T. compound (Sakura Finetek USA Inc., Torrance CA, USA) in liquid nitrogen-cooled isopentane and saved at −80°C for histological analysis. A PowerLab/LabChart Software system (ADInstruments, Colorado Springs, CO, USA) was used to store and analyze force data. Muscle force is reported as absolute force (mN) and force normalized to the muscle physiological cross-sectional area (N/cm^2^) via the following formula:

(force (mN) × muscle length (mm) × 1.06 (muscle density))/=(muscle weight (mg) × 0.1).

### Cardiac RNA isolation and real-time RT-PCR

Hearts were isolated from 18 mo and 22 mo old VWR and CTRL mice and placed in a dish containing Hank’s balanced salt solution. Next, the heart was cut transversely and blood and excess tissues were removed. The heart was then weighed for determination of heart weight to body weight ratio (HW/BW), flash frozen in liquid nitrogen and stored at −80°C. Total RNA was extracted from frozen heart samples for *alpha-myosin heavy chain* (*α-MHC*, *Myh6*) and *beta-MHC (β-MHC, Myh7)* gene expression using a RNeasy Fibrous Tissue Mini Kit (Qiagen, Valencia, CA, USA) and further cleaned using a RNeasy MinElute Cleanup Kit (Qiagen). Premade TaqMan primer/probe sets specific for mouse are as follows: *β-actin* (Mm01205647_g1), *α-MHC* (Mm00440359_m1) and *β-MHC* (Mm00600555_m1) (Applied Biosystems, Foster City, CA, USA). Real-time RT-PCR was performed with an ABI One Step RT-PCR kit using a Rotor-Gene 6000 real-time PCR system (Qiagen). The cycle threshold (Ct) value was determined for each gene, and the relative gene expression levels of *α-MHC* and *β-MHC* were calculated using 2^–ΔCT^ values, with β-actin as the housekeeping gene.

### Assessment of skeletal muscle fiber type and cross-sectional area (CSA)

Muscles were retrieved from the contractility experiments, excess Ringer’s solution removed and muscle placed in Tissue-Tek O.C.T. compound before transfer to a Cryomold. The muscle was oriented into its natural shape by gently pulling the ends of tendon and the Cryomold was snap frozen in isopentane in liquid nitrogen. 7 μm thick transverse cryosections were cut using a Leica CM3050S cryomicrotome (Leica Microsystems, Wetzlar, Germany) from four standardized levels through the muscle, at 750 μm intervals between levels, with three sections cut per level.

Muscle fiber typing was determined using a 4-color immunofluorescent staining method using antibodies specific for myosin heavy chains (MyHC) corresponding to the different fiber types. These antibodies included: Mouse IgG2b BA-D5 specific for MyHC I (1:100, Developmental Studies Hybridoma Bank (DSHB), Iowa City, IA, USA), mouse IgG1 SC-71 specific for MyHC IIa (1:100, DSHB), mouse IgM BF-F3 specific for MyHC IIb (1:20, DSHB). In addition, we used a rabbit anti-dystrophin antibody (1:400, ab15277, Abcam, Cambridge, England) to label the border of each fiber. The muscle cryosections were air dried for 5 min and fixed with acetone for 5 min at room temperature. They were then blocked to eliminate non-specific binding in M.O.M^®^ Blocking Reagent (1:25, MKB-2213, Vector Laboratories, Inc., Burlingame, CA, USA) for 1 hr at room temperature. The sections were then incubated with primary antibodies specific for each MyHC as described above, along with anti-dystrophin antibody overnight at 4°C. After washing in phosphate-buffered saline (PBS), the sections were incubated with the secondary antibodies for 2 hr at room temperature. These included: an Alexa Fluor 647 goat anti-mouse IgG2b secondary antibody (A21242, Invitrogen, Carlsbad, CA, USA) for BA-D5, an Alexa Fluor 488 goat anti-mouse IgG1 secondary antibody (A21121, Invitrogen) for SC-71, an Alexa Fluor 594 goat anti-mouse IgM secondary antibody (A21044, Invitrogen) for BF-F3, an Alexa Fluor 350 goat anti-rabbit IgG (H+L) secondary antibody (A11046, Invitrogen) for dystrophin. After further washing in PBS and post-fixation with methanol, sections were coverslip mounted using vector shield mounting media (H-1000, Vector Laboratories, Inc.). For analysis of fiber diameter, sections were immunostained singly with the rabbit anti-dystrophin antibody as above, but using a CF555 goat anti-rabbit IgG (H+L) secondary antibody (20232, Biotium, Fremont, CA, USA).

For fiber typing, the fluorescent images were acquired using a Keyence BZ-X810 microscope using a 10 × .45NA objective and excitation and emission filters appropriate for each fluorescence dye. Pseudocoloring and merging of multiplexed images were performed by a BZ-X800 analyzer. To quantify the number of each fiber type, the Multi Point counting tool was used in the Image J software [110]. The skeletal muscle fibers that were not stained with any antibodies were counted as Type IIX. For analysis of the muscle fiber cross sectional area (CSA) and diameter, the sections were observed under a Lionheart FX microscope (BioTek, Winooski, VT, USA) using a 40× objective and thresholded images were quantified using the Gen5 data analysis software (BioTek). Two serial images were examined per sample and more than 500 muscle fibers were measured per serial image.

### Adipose analysis by histomorphometry

The distal femur was fixed in 4% paraformaldehyde in PBS, pH 7.4 for 24 hr, then transferred to 70% ethanol. Undecalcified distal femurs were then dehydrated in a graded series of alcohols. They were then infiltrated with acetone, then 1:1 followed by 1:2 acetone:methyl methacrylate infiltration solution [84% methyl methacrylate (MMA), 14% dibutyl phthalate, 1% polyethylene glycol, 0.7% benzoyl peroxide], and then two changes of 100% MMA infiltration solution (solutions changed daily). Samples were then embedded in a 20 ml glass vial containing 5 ml pre-polymerized base by adding 10 ml freshly made MMA embedding solution [as above for the infiltration solution but with benzoyl peroxide reduced to 0.4% and with 0.33% N,N-dimethyl p-toluidine]. Polymerization was done at −20°C for 3–5 days.

Blocks were trimmed with a Buehler ISOMET 1000 precision saw, then 5 μm longitudinal sections were cut on a Thermo Scientific Microm HM355S microtome, using a Dorn and Hart Microedge Tungsten Carbide D profile knife. After the sections were deplasticized and rehydrated they were stained with Goldner’s stain using standard histological procedures and marrow adipose tissue was quantified using a 10× objective with a Nikon E800 microscope (Nikon Instruments, Inc. Melville NY, USA) and SONY Exwave HAD camera (Sony Corp., NY, USA) interfaced with an Osteomeasure bone histomorphometry system (OsteoMetrics Decatur, GA, USA). Bone marrow adipose percent area was measured in the entire metaphyseal region below the growth plate and extending distally 550 μm below the lowest point of the growth plate. Values were averaged from 3 non-adjacent sections per animal for each data point.

### Cell death assay *in vitro*

#### Preparation of muscle conditioned media (CM)

Muscle CM were prepared from intact EDL and soleus muscles dissected from the 18 and 22 mo old C57BL/6 female mice. Muscles were placed inside Contractility Chambers containing 20 mL of Ringer’s solution (142 mM NaCl; 5 mM KCl; 1.8 mM MgCl2; 10 mM HEPES; 2.5 mM CaCl2; 10 mM glucose; pH 7.4) using an eight-chamber system driven by an ADI-PowerLab Software. The experimental conditions previously defined [19, 111] were used in this study with some modifications. Briefly, static CM was obtained by placing muscles in the chamber without stimulation for 30 min. Next, the optimal length of the muscle allowing it to achieve maximal force was determined by the length-force relationship. Muscles were then equilibrated with stimulatory trains of 500 ms, 90 Hz, repeated every minute for 30 min to collect the contracted muscle CM.

#### Quantification of cell death

The osteocyte-like MLO-Y4 cell line, derived from murine long bone, was used as an *in vitro* osteocyte model [112]. MLO-Y4 cells were maintained on rat tail collagen type I (Thermo Fisher Scientific, Waltham, MA, USA)-coated plates in α-MEM supplemented with 2.5 % fetal bovine serum (FBS), 2.5% calf serum (CS) in a 5% CO_2_ incubator at 37°C. MLO-Y4 cells were plated at 1 × 10^4^/cm^2^ on a type I collagen-coated 96-well plate with 6 wells for each experimental condition. Cells were pretreated with 10% muscle CM in 1% FBS/ 1% CS/α-MEM for 24 hr, followed by treatment with 0.3 mM hydrogen peroxide (H1009, Sigma, St. Louis, MO) for 3–4 hr in 1% FBS/1% CS/α-MEM to induce cell death. Cells were stained with 2 μM ethidium homodimer 1 (E1169, Invitrogen) and 5 μg/mL Hoechst 33342 (H1399, Invitrogen) in media for 30 min and analyzed on a Lionheart FX microscope to detect dead cells. Images were acquired using a 4× objective under epifluorescence illumination and thresholded images were quantified using the Gen5 data analysis software. Cell death was calculated as ethidium homodimer 1 positive cells divided by the total number of cells stained with Hoechst 33342 as a nuclear counterstain. Data are presented as fold change compared to the level of cell death in the hydrogen peroxide-treated group.

### Evaluation of osteocyte connectivity

#### 3D Multiplexed confocal imaging

The left femurs were fixed in 4% paraformaldehyde in PBS, pH 7.4 at 4°C for 48 hr with gentle shaking. After fixation femurs were decalcified in 10% EDTA, pH 7.4 for 2 weeks, then equilibrated in 15% sucrose in PBS followed by 30% sucrose in PBS before embedding in Tissue-Tek O.C.T. compound and freezing overnight at −80°C. 50 μm transverse sections were cut using the Leica CM3050S cryomicrotome from the midshaft region distal to the third trochanter. The thick sections were stained en block by incubation in 165 nM Alexafluor488-phalloidin (Thermo Fisher Scientific) in PBS overnight followed by three PBS washes and then a 30 min incubation in 4 μg/ml DAPI (4’,6-diamidino-2-phenylindole, Thermo Fisher Scientific) in PBS. For full details of our published multiplexed staining methods, see [113]. Stained sections were coverslip mounted in 1:1 glycerol: PBS with 1 mM MgCl_2_. Two mounted sections per animal were imaged on a Leica TCS Sp5 II laser scanning confocal microscope (Leica Microsystems, Wetzlar, Germany) in resonant scanner mode. Detailed Z-stacks of 250–350 z-planes were obtained with a 100× oil objective (NA 1.44) using a zoom of 1.7 with a 0.13 μm step size from three standardized regions in the midshaft sections [45]. For visualization of phalloidin, 488 nm laser excitation was used with an emission collection window of 493–580 nm acquired together with a brightfield image. DAPI images of the nuclei were acquired in a separate scan using 405 nm laser excitation and a collection window of 410–480 nm.

#### Image quantification

To quantify osteocyte density phalloidin/DAPI stained 100× image stacks with 250 slices (32.5 μm tissue depth) were used to count the number of osteocytes in the 272,217 μm^3^ volume using the Image J cell counting plugin. These results were averaged for the six imaging fields to obtain the mean number of osteocytes per mm^3^ of bone for each animal. To quantify the number of dendrites per osteocyte, the 100× stacks of phalloidin labeled sections were contrasted in ImageJ. Using the Image J Cell Counting plugin, dendrites were counted by scrolling through the Z stack and counting each dendrite at the point where it sprouted from the cell body. At least 30 complete osteocytes were counted per animal to obtain the average number of dendrites per osteocyte for each animal. Full details of the osteocyte density and dendrite counting methods were published previously [45].

### Quantification of osteocyte apoptosis

#### Tissue collection and processing

Right femurs were fixed in 4% paraformaldehyde as described above. They were cut into halves by diamond saw (NSK MIO E 120 Micromotor) and the proximal halves were demineralized in 10% EDTA/PBS pH 7.4 as described above. The bones were subsequently washed with PBS, dehydrated in graded alcohols from 70% ethanol to absolute alcohol for 1–2 hr each, cleared with xylene, and then paraffin embedded. All steps were performed in a SAKURA Tissue-Tek VIP6 auto tissue processor. 5 μM paraffin sections were cut, dewaxed and rehydrated. Osteocyte apoptosis was detected by TdT-mediated dUTP nick-end labeling (TUNEL) using a commercial kit (A049, ABP Biosciences, Rockville, MD, USA). Sections were treated with 20 μg/mL Proteinase K in PBS for 30 min at room temperature for antigen retrieval, then incubated with 3% H_2_O_2_ for 10 min at room temperature to inactivate endogenous peroxide, and then with 20 μl TdT reaction cocktail per section at 37°C for 2 hr in a humid chamber. The cocktail without TdT Enzyme was added as the negative control. The Vector PK-4000 ABC detection system (Vector Laboratories, Inc.) was used to visualize signals. The TUNEL reaction was counterstained using 0.5% methyl green.

#### Quantification of apoptosis

Osteocytes were counted through the entire cortical width for each of six sampling regions in the femur sagittal sections. Osteocytes were classified into four groups: Category I (TUNEL-negative, Live), Category II (stained positive for methyl green and TUNEL, Dying), Category III (TUNEL-positive, Apoptotic), and Category IV (Empty lacuna). Each type of osteocyte was expressed as a percentage of the total number of lacunae. These results were averaged for the six imaging fields to obtain the mean percentage of each group for each animal.

### Micro-CT analysis

Mouse femurs were scanned in the Bruker Skyscan 1174 (Billerica, MA, USA) at a nominal resolution of 9.6 microns employing an aluminum filter 0.5 mm thick and an applied x-ray tube voltage of 50 kV. Camera pixel binning was not applied. The scan orbit was 180/360 degrees with a rotation step of 0.4 degrees. Reconstruction was carried out with a modified Feldkamp algorithm using the SkyScan^™^ NRecon software accelerated by GPU3. Gaussian smoothing, ring artifact reduction and beam hardening correction were applied.

The stack of the reconstructed images was analyzed using CTAn software. Fifty slices (0.5 mm) from the mid diaphysis were selected for each femur to calculate the cortical bone parameters. The minimum threshold was set to 80 and the maximum was set to 255 for all the bones. One hundred slices (1 mm) from the growth plate of distal femur were selected to calculate the trabecular bone parameters.

### Bone static histomorphometry

Bone static histomorphometry was performed to obtain mechanistic insight into the alterations of cortical bone in response to VWR. Fixed and undecalcified distal femurs were embedded in MMA resin, and 5-μm-thick longitudinal sections were deplasticized and stained with TRAP (tartrate-resistant acid phosphatase), 2% naphthol AS-BI phosphate as a substrate, with the reaction of the product with 5% pararosaniline treated with 4% sodium nitrite, and counterstained with 0.1% toluidine blue. Histomorphometric analysis of the distal femoral midshaft region was performed using the OsteoMeasure bone histomorphometry system interfaced with a Nikon E800 microscope and SONY Exwave HAD camera. Images were captured and quantified in the midshaft region, ~1 mm in length, 3 mm distal from the lowest point of the growth plate. For each data point, values for osteoclast number and surface on the endocortical surfaces were averaged from 3 non-adjacent sections per animal. In addition, von Kossa tetrachrome staining was performed using standard histological procedures with 5% silver nitrate and 3% tetrachrome solution to visualize osteoblasts and mineral. However, quantitative data on osteoblasts was not performed, but representative photomicrographs are provided as osteoblasts in aged animals have different morphology, no longer plump and polygonal, as seen in young or middle-aged animals.

The same sections used for bone marrow adipose analysis were also measured for cortical porosity. Images were captured and quantified in the midshaft region of the femurs, 3 mm in length, 3 mm distal from the lowest point of the growth plate using the OsteoMeasure bone histomorphometry system. All the porosities excluded osteocyte lacunae.

### Three-point bending test

Three-point bending was performed on tibias that had been freshly frozen in PBS-soaked gauze at sacrifice. The samples were thawed at least half an hour before testing was started. All tests were performed on a Bose 3230 Dynamic Loading system using a 225 N load cell and Wintest 4.0 software. Prior to testing the length, minimum and maximum diameter and weight were measured for each sample. Tibias were tested while keeping the posterior-anterior axis in the loading direction and the lateral-medial axis in the bending direction. All samples were centered on the supports and the force was applied vertically to the midshaft at a constant speed of 0.1 mm/sec until fracture occurred. Displacement and load data were collected for biomechanical analysis to obtain bone properties using an ImageJ plugin, BoneJ [114].

### Nanoindentation procedure

#### Mouse bone sample preparation process

Femurs were cut into 3 mm transverse sections using a low-speed diamond saw and polished using a series of diamond pastes (6, 1 and 0.25 μm) to remove any scratches.

#### Indentation process

A Hysitron TriboScope AFM/Nanoindenter fitted with a standard three-sided pyramidal Berkovich probe was used to perform the testing of the polished bone sections. Triboscan software was used to run the equipment and perform the analysis of the data. Prior to testing the bone samples a quartz calibration standard was indented to ensure the equipment/probe were performing within specifications. After a z-axis calibration (in-air) and finding a region without any scratches/artifacts the indentation procedure was performed using the following loading profile: maximum force of 1000 μN, a segment time of 5 seconds (200 μN per second), 3 seconds of indent and then 5 seconds of de-load. The subsequent load versus displacement plots were used to calculate both the hardness and elastic modulus of the bone samples.

### Statistical analysis

Longitudinal outcomes were analyzed by repeated measure ANOVA. For comparisons between two groups of normally distributed data the student’s *t*-test was used. For heart size, muscle force, size, and kinetics data there are significant previously reported data indicating changes after VWR, therefore a one-tailed *t*-test was used. We also used a one-tailed *t*-test for analyzing bone-related parameters except for bone μCT data analysis with a two-tailed *t-*test based on our working hypotheses. Mann Whitney test was used to analyze two groups of datasets that were not normally distributed. For statistical comparisons between more than two groups of datasets, one-way analysis of variance (ANOVA) was used followed by Tukey’s post hoc test. Kruskal Wallis was used to analyze datasets that were not normally distributed with Dunn’s post hoc test. Two-way ANOVA with Bonferroni was used to compare the mean differences between groups split by two independent variables. The Pearson Correlation analysis was performed to measure the strength of the linear relationship between two variables. The interaction analysis among body weight, VWR and age was performed by Multivariable Regression. A *p*-value <0.05 was considered statistically significant. They were labeled as ^*^: *p* < 0.05, ^**^: *p* < 0.01, ^***^: *p* < 0.001 and ^****^: *p* < 0.0001.

## Supplementary Materials

Supplementary Figures

Supplementary Tables
