# Supplementary material for: Body weight influences musculoskeletal adaptation to long-term voluntary wheel running during aging in female mice
Source: Aging (Albany NY). 2022 Nov 18;15(2):308–52. doi: 10.18632/aging.204390 (PMC9925690; doi:10.18632/aging.204390)
Supplement: Supplementary Figures [file aging-15-204390-s001.pdf]

SUPPLEMENTARY FIGURES

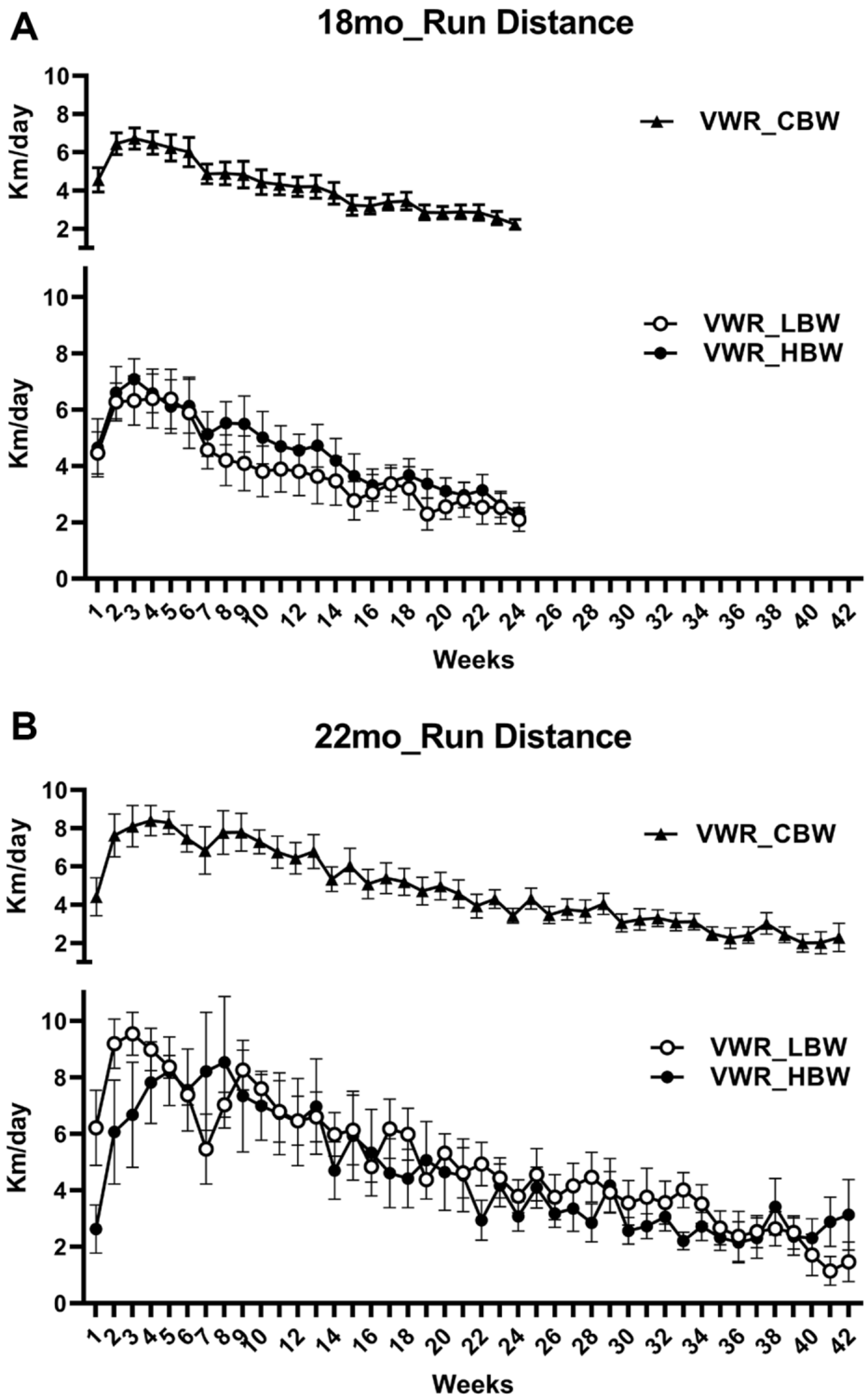

Supplementary Figure 1. Wheel running profile. The running distance of 18 mo (**A**) and 22 mo mice (**B**). No significant differences were found.

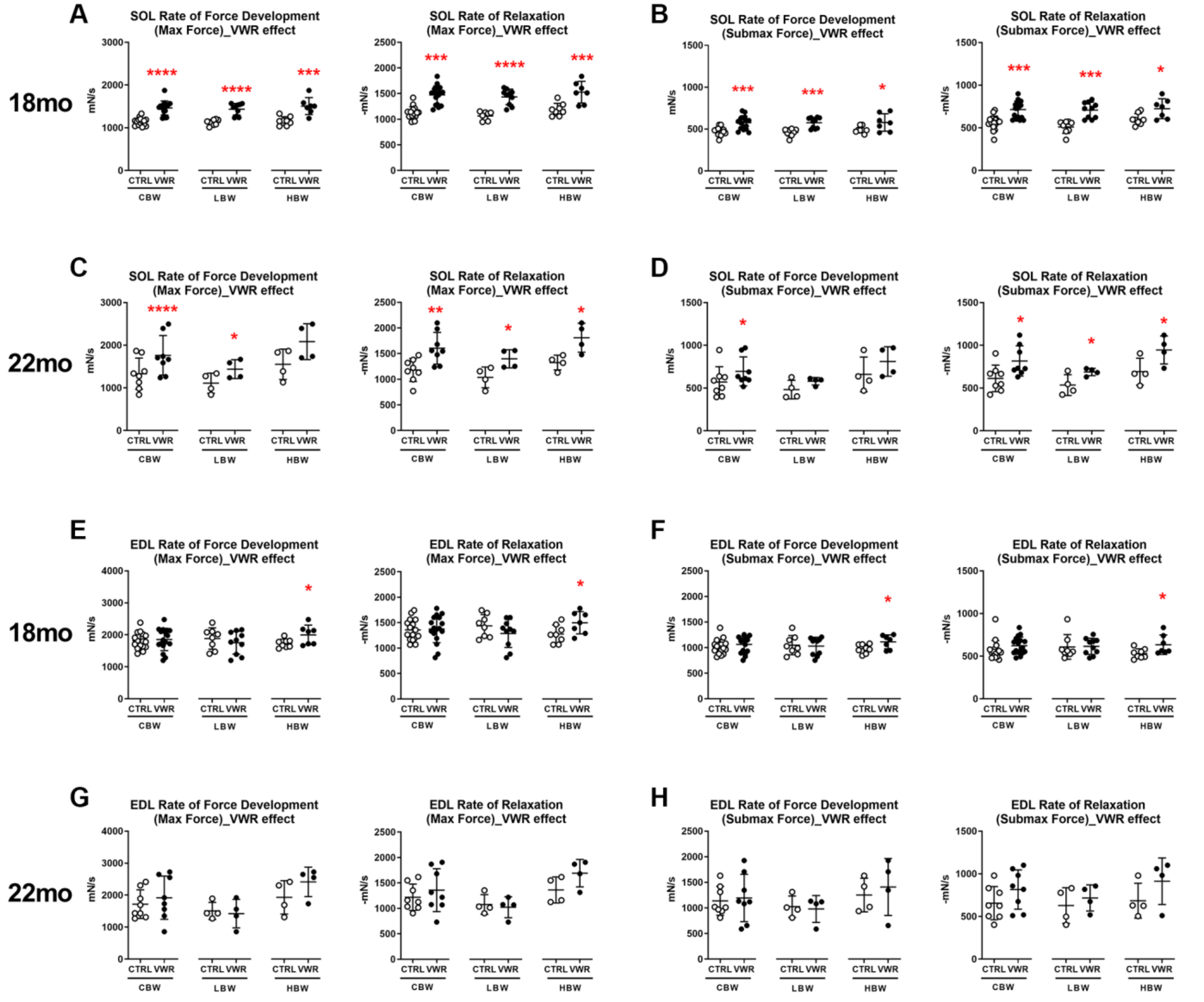

Supplementary Figure 2. The effect of long-term endurance exercise on soleus and EDL skeletal muscle kinetic properties of contraction. 6 mo and 10 mo endurance exercise increased the rates of force development and relaxation of soleus muscle contractions. 6 mo endurance exercise increased the rates of force development and relaxation of EDL muscle contractions in HBW mice only. The rate of soleus muscle force development (left) and the rate of relaxation (right) in 18 mo mice for contractions at (**A**) maximal force and (**B**) submaximal force. The rate of force development (left) and the rate of relaxation (right) in 22 mo mice for contractions at (**C**) maximal force and (**D**) submaximal force. The rate of EDL muscle force development (left) and the rate of relaxation (right) in 18 mo mice for contractions at (**E**) maximal force and (**F**) submaximal force. The rate of force development (left) and the rate of relaxation (right) in 22 mo mice for contractions at (**G**) maximal force and (**H**) submaximal force. *: $p < 0.05$, **: $p < 0.01$, ***: $p < 0.001$ and ****: $p < 0.0001$. VWR vs. CTRL mice. Abbreviations: CTRL: control group; VWR: voluntary wheel running group; CBW: Combined groups (18 mo: *n* = 16 CTRL, *n* = 17 VWR; 22 mo: *n* = 8 CTRL, *n* = 8 VWR); LBW: Low body weight group (18 mo: *n* = 8 CTRL, *n* = 10 VWR; 22 mo: *n* = 4 CTRL, *n* = 4 VWR); HBW: High body weight group (18 mo: *n* = 8 CTRL, *n* = 7 VWR; 22 mo: *n* = 4 CTRL, *n* = 4 VWR).

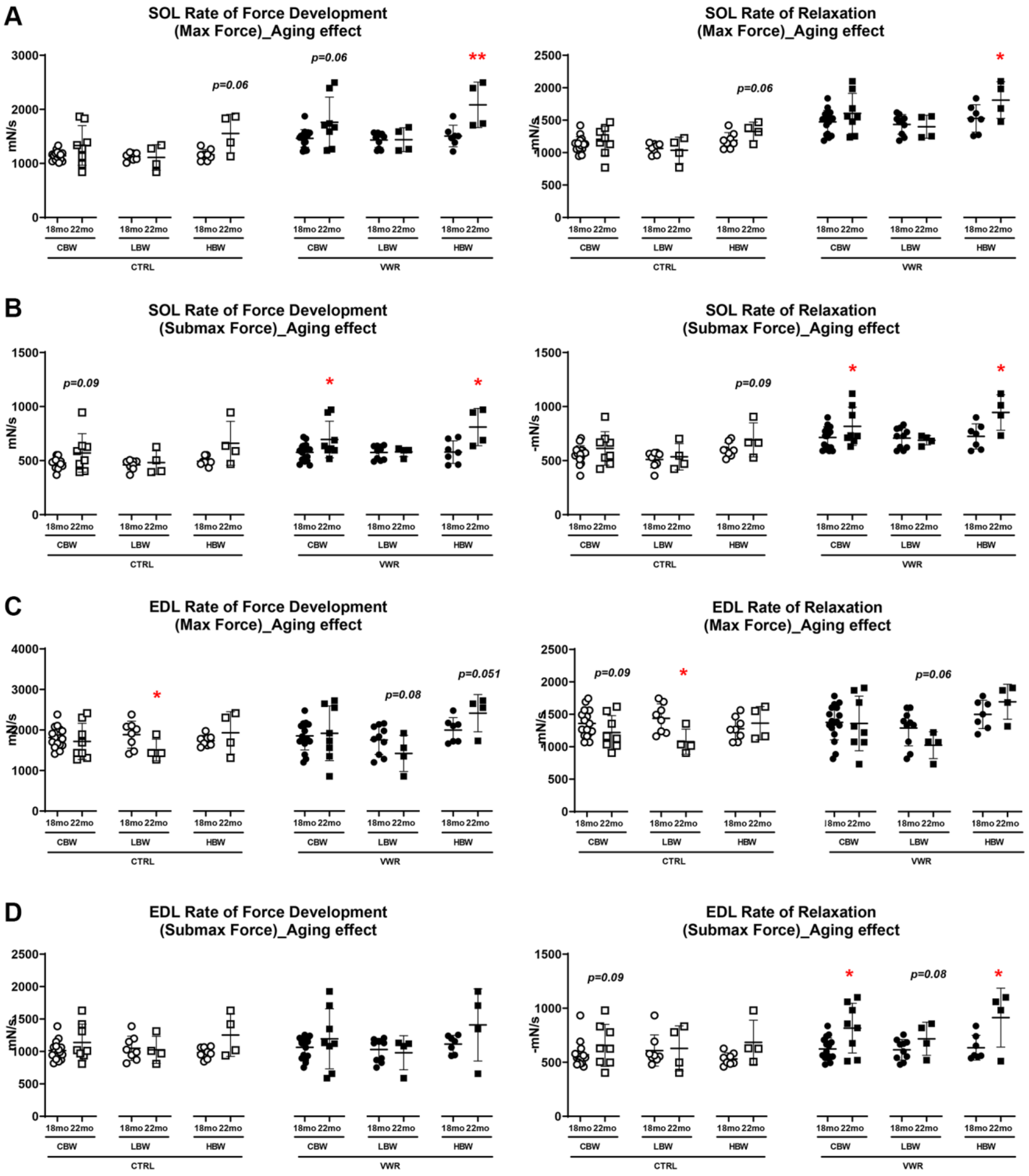

Supplementary Figure 3. Aging effects on soleus and EDL skeletal muscle kinetic properties of contraction. (**A**) The rate of soleus muscle force development (left) and the rate of relaxation (right) for maximal contractile force, (**B**) the rate of soleus muscle force development (left) and the rate of relaxation (right) for submaximal contractile force, (**C**) The rate of EDL muscle force development (left) and the rate of relaxation (right) for maximal contractile force and (**D**) the rate of EDL muscle force development (left) and the rate of relaxation (right) for submaximal contractile force in 18 mo and 22 mo LBW and HBW mice. *: $p < 0.05$, **: $p < 0.01$. VWR vs. CTRL mice. Abbreviations: CTRL: control group; VWR: voluntary wheel running group; CBW: Combined groups (18 mo: $n = 16$ CTRL, $n = 17$ VWR; 22 mo: $n = 8$ CTRL, $n = 8$ VWR); LBW: Low body weight group (18 mo: $n = 8$ CTRL, $n = 10$ VWR; 22 mo: $n = 4$ CTRL, $n = 4$ VWR); HBW: High body weight group (18 mo: $n = 8$ CTRL, $n = 7$ VWR; 22 mo: $n = 4$ CTRL, $n = 4$ VWR).

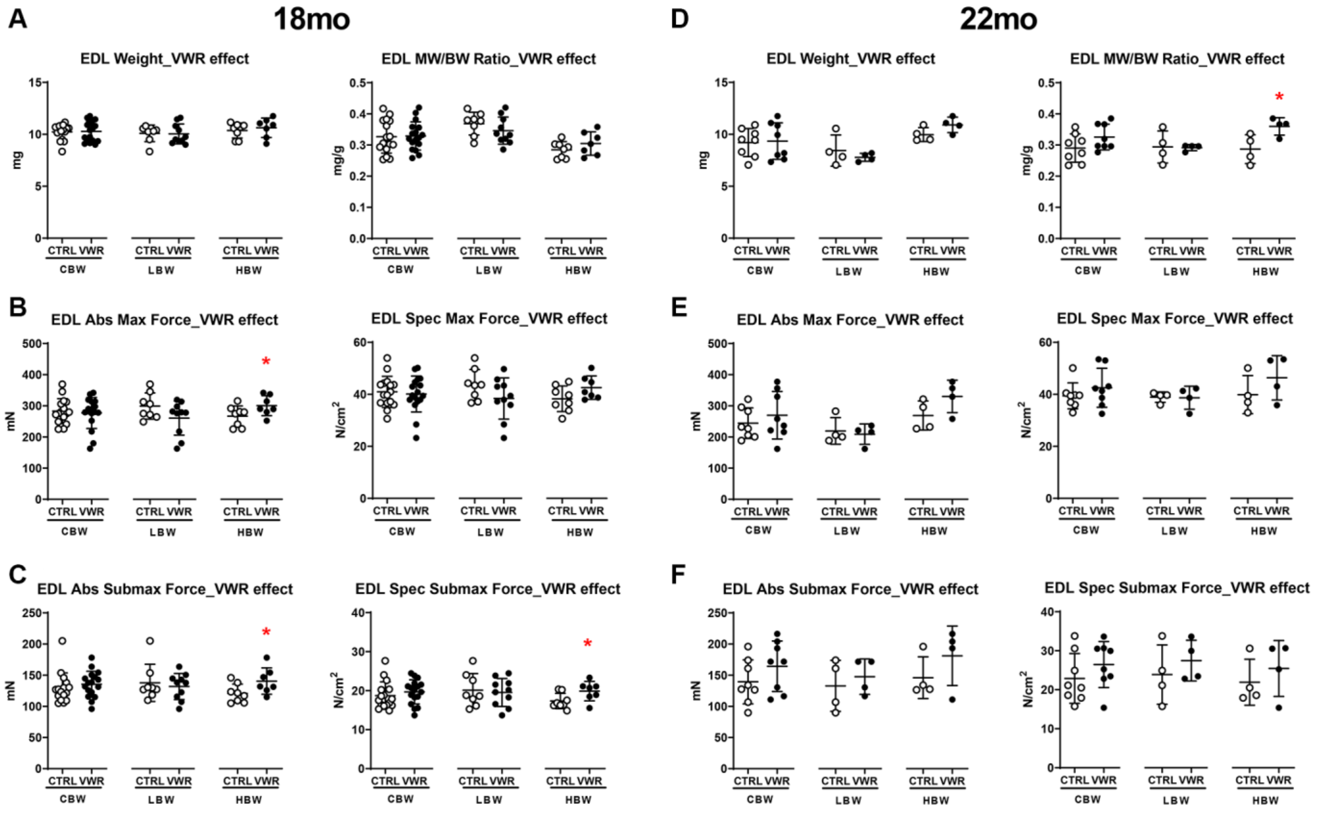

Supplementary Figure 4. The effect of long-term endurance exercise on EDL skeletal muscle weights and contractile force. Endurance exercise increased EDL muscle weight normalized to body weight in 22 mo old mice, while endurance exercise improved both absolute and specific submaximal contractile force in the 18 mo old HBW group only. (**A**) EDL muscle weight (left) and muscle weight/body weight (MW/BW) ratio (right) for 18 mo mice. (**B**) EDL muscle absolute maximal force (left) and specific maximal force (right) in 18 mo mice. (**C**) EDL muscle absolute submaximal force (left) and specific submaximal force (right) in 18 mo mice. (**D**) EDL muscle weight (left) and muscle weight/body weight (MW/BW) ratio (right) for 22 mo mice. (**E**) EDL muscle absolute maximal force (left) and specific maximal force (right) in 22 mo mice. (**F**) EDL muscle absolute submaximal force (left) and specific submaximal force (right) in 22 mo mice. *: $p < 0.05$. VWR vs. CTRL mice. Abbreviations: CTRL: control group; VWR: voluntary wheel running group; CBW: Combined groups (18 mo: n = 16 CTRL, n = 17 VWR; 22 mo: n = 8 CTRL, n = 8 VWR); LBW: Low body weight group (18 mo: n = 8 CTRL, n = 10 VWR; 22 mo: n = 4 CTRL, n = 4 VWR); HBW: High body weight group (18 mo: n = 8 CTRL, n = 7 VWR; 22 mo: n = 4 CTRL, n = 4 VWR).

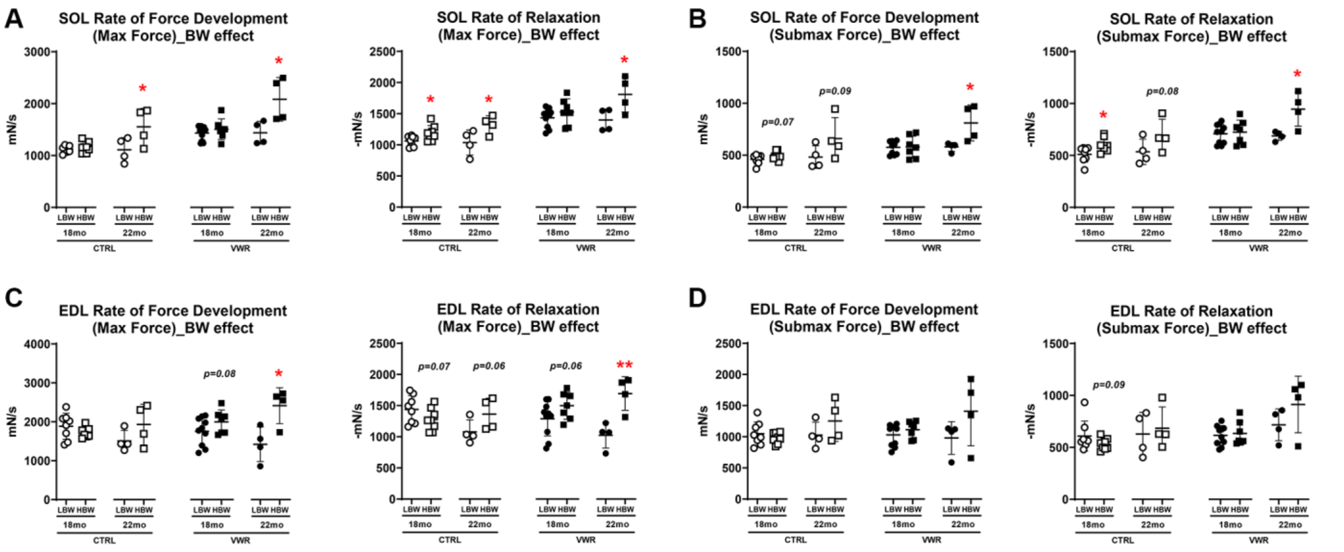

Supplementary Figure 5. Body weight effects on soleus and EDL skeletal muscle kinetic properties of contraction. (**A**) The rate of soleus muscle force development (left) and the rate of relaxation (right) for maximal contractile force, (**B**) the rate of soleus muscle force development (left) and the rate of relaxation (right) for submaximal contractile force, (**C**) The rate of EDL muscle force development (left) and the rate of relaxation (right) for maximal contractile force and (**D**) the rate of EDL muscle force development (left) and the rate of relaxation (right) for submaximal contractile force in 18 mo and 22 mo LBW and HBW mice. *: $p < 0.05$, **: $p < 0.01$. VWR vs. CTRL mice. Abbreviations: CTRL: control group; VWR: voluntary wheel running group; LBW: Low body weight group (18 mo: *n* = 8 CTRL, *n* = 10 VWR; 22 mo: *n* = 4 CTRL, *n* = 4 VWR); HBW: High body weight group (18 mo: *n* = 8 CTRL, *n* = 7 VWR; 22 mo: *n* = 4 CTRL, *n* = 4 VWR).

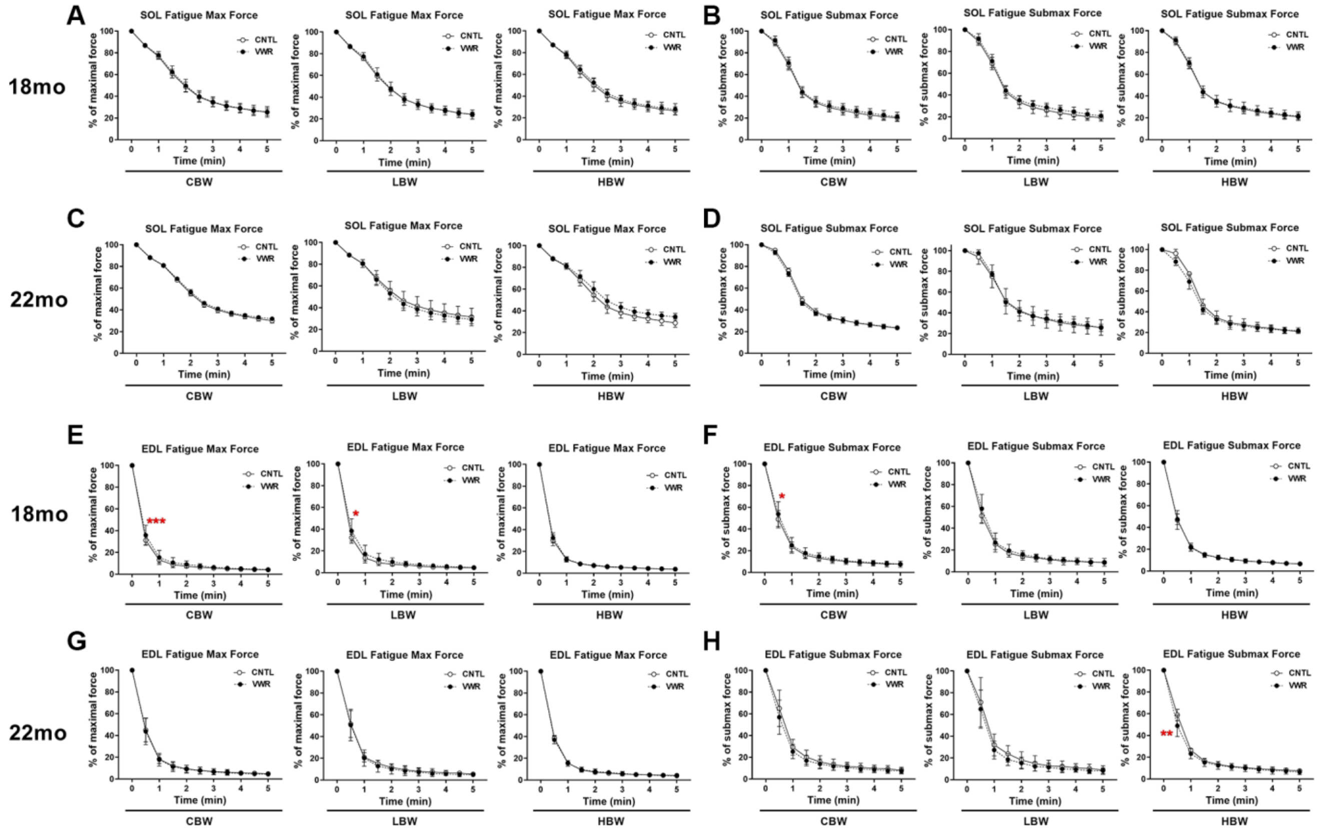

Supplementary Figure 6. The effect of long-term endurance exercise on soleus and EDL skeletal muscle fatigue profile. 6 mo and 10 mo endurance exercise did not have an effect on soleus muscle resistance to fatigue. In EDL muscle, 6 mo but not 10 mo endurance exercise improved EDL muscle resistance to early fatigue. Muscle force during the fatiguing protocol expressed as a percentage of initial force for CBW (left), LBW (middle) and HBW (right) groups for: (**A**) soleus muscle maximal force in 18 mo mice, (**B**) soleus muscle submaximal force in 18 mo mice, (**C**) soleus muscle maximal force in 22 mo mice, and (**D**) soleus muscle submaximal force in 22 mo mice, (**E**) EDL muscle maximal force in 18 mo mice, (**F**) EDL muscle submaximal force in 18 mo mice, (**G**) EDL muscle maximal force in 22 mo mice, and (**H**) EDL muscle submaximal force in 22 mo mice. *: $p < 0.05$ and ***: $p < 0.001$. VWR vs. CTRL mice. Abbreviations: CTRL: control group; VWR: voluntary wheel running group; CBW: Combined groups (18 mo: n = 15–16 CTRL, n = 16–17 VWR; 22 mo: n = 8 CTRL, n = 8 VWR); LBW: Low body weight group (18 mo: n = 7–8 CTRL, n = 9–10 VWR; 22 mo: n = 4 CTRL, n = 4 VWR); HBW: High body weight group (18 mo: n = 8 CTRL, n = 7 VWR; 22 mo: n = 4 CTRL, n = 4 VWR).

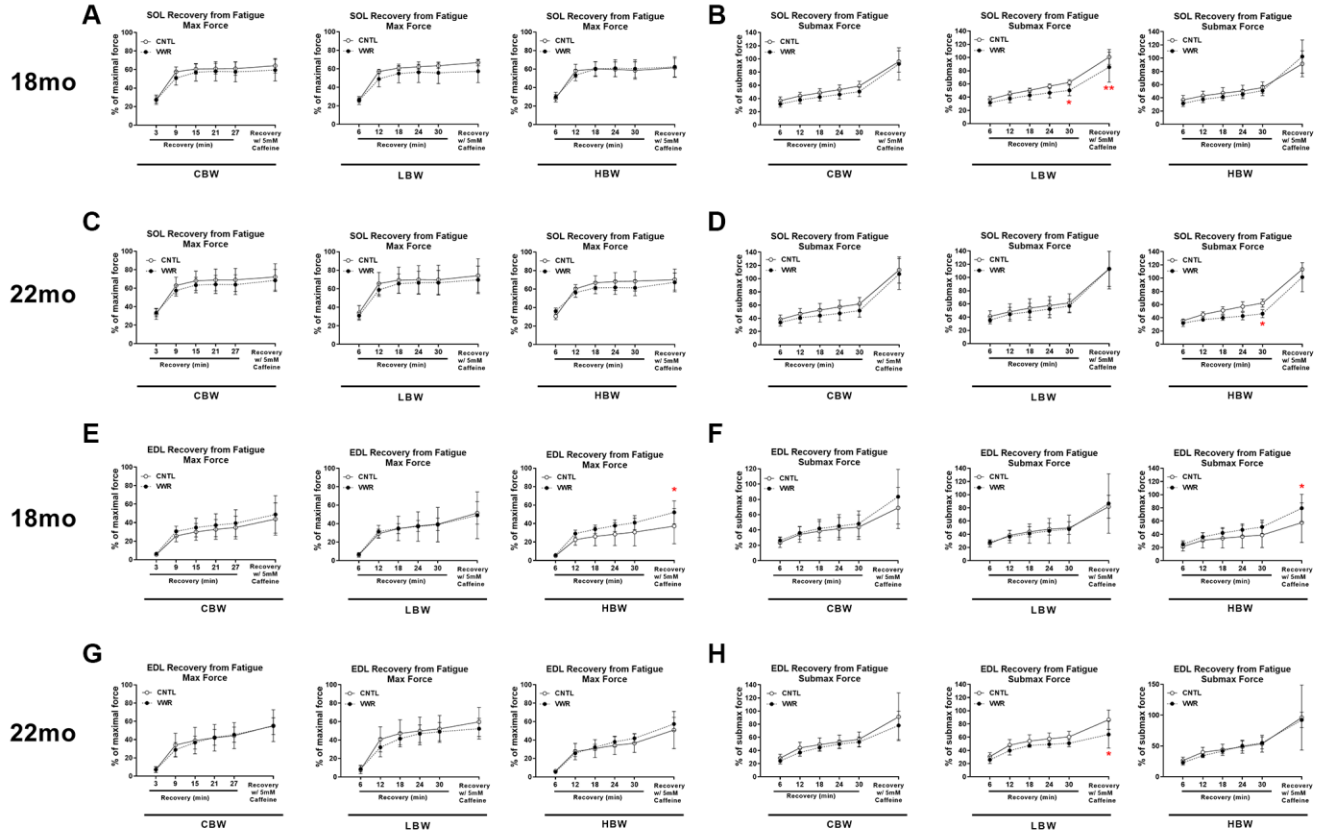

Supplementary Figure 7. The effect of long-term endurance exercise on soleus and EDL skeletal muscle recovery from fatigue. 6 mo and 10 mo endurance exercise had little impact on soleus muscle force recovery following fatigue. In EDL, 6 mo but not 10 mo endurance exercise enhanced recovery from fatigue after caffeine administration in HBW only. Soleus muscle force during the recovery period expressed as a percentage of force before fatigue in CBW (left), LBW (middle) and HBW (right) groups for (**A**) maximal force in 18 mo mice, (**B**) submaximal force in 18 mo mice, (**C**) maximal force in 22 mo mice, and (**D**) submaximal force in 22 mo mice. EDL muscle force during the recovery period expressed as a percentage of force before fatigue in CBW (left), LBW (middle) and HBW (right) groups for (**E**) maximal force in 18 mo mice, (**F**) submaximal force in 18 mo mice, (**G**) maximal force in 22 mo mice, and (**H**) submaximal force in 22 mo mice. *: $p < 0.05$ and **: $p < 0.01$. VWR vs. CTRL mice. Abbreviations: CTRL: control group; VWR: voluntary wheel running group; CBW: Combined groups (18 mo: *n* = 15–16 CTRL, *n* = 16–17 VWR; 22 mo: *n* = 8 CTRL, *n* = 8 VWR); LBW: Low body weight group (18 mo: *n* = 7–8 CTRL, *n* = 9–10 VWR; 22 mo: *n* = 4 CTRL, *n* = 4 VWR); HBW: High body weight group (18 mo: *n* = 8 CTRL, *n* = 7 VWR; 22 mo: *n* = 4 CTRL, *n* = 4 VWR).

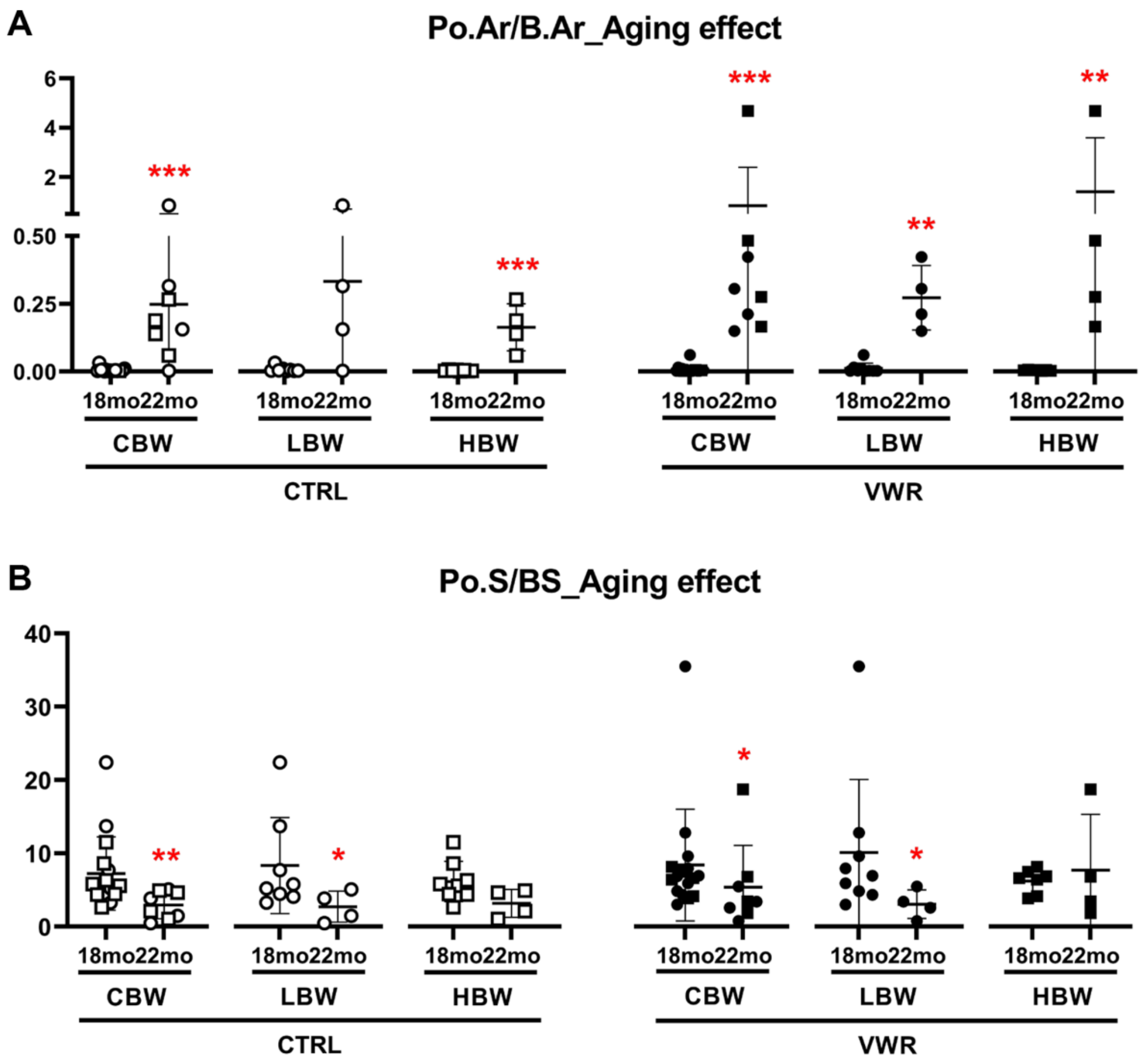

Supplementary Figure 8. Cortical porosity was increased with aging. Aging effects were observed in the cortical porosity area in 22 mo CTRL/HBW and both LBW and HBW subgroups of 22 mo VWR mice, and porosity surface in LBW subgroups in 22 mo CTRL and VWR mice. (**A**) Porosity area Po.Ar/B.Ar, and (**B**) Porosity surface Po.S/B.S were measured using the OsteoMeasure bone histomorphometry system. *: $p < 0.05$, **: $p < 0.01$, and ***: $p < 0.001$, 22 mo vs. 18 mo mice. Abbreviations: CTRL: control group; VWR: voluntary wheel running group; CBW: Combined groups (18 mo: $n = 16$ CTRL, $n = 16$ VWR; 22 mo: $n = 8$ CTRL, $n = 8$ VWR); LBW: Low body weight group (18 mo: $n = 8$ CTRL, $n = 9$ VWR; 22 mo: $n = 4$ CTRL, $n = 4$ VWR); HBW: High body weight group (18 mo: $n = 8$ CTRL, $n = 7$ VWR; 22 mo: $n = 4$ CTRL, $n = 4$ VWR).

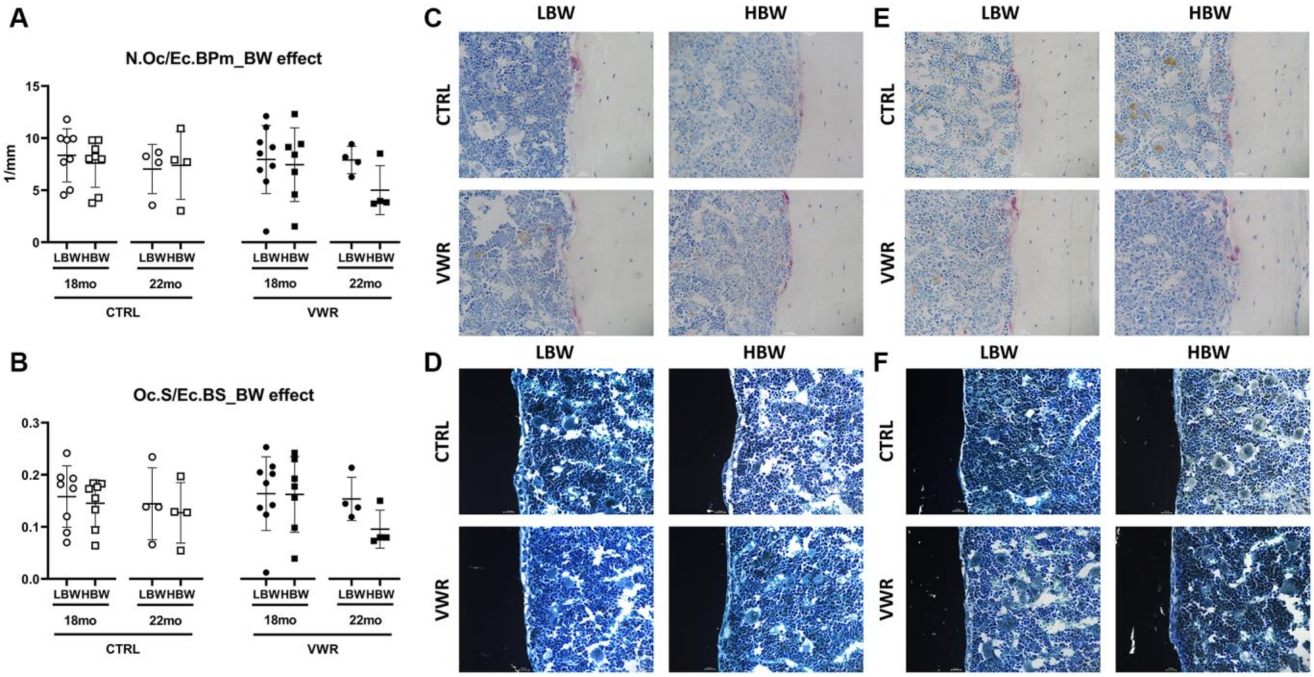

Supplementary Figure 9. No significant differences in osteoclast number and surface between any group. The effect of body weight (BW) on osteoclast number and surface on the femoral midshaft endocortical surface in two different age groups, 18 mo and 22 mo mice, is shown. (**A**) Osteoclast number N.Oc/Ec.BPm, and (**B**) Osteoclast surface Oc.S/Ec.BS were measured using the OsteoMeasure bone histomorphometry system. No significance was observed. Abbreviations: CTRL: control group; VWR: voluntary wheel running group; LBW: Low body weight group (18 mo: *n* = 8 CTRL, *n* = 9 VWR; 22 mo: *n* = 4 CTRL, *n* = 4 VWR); HBW: High body weight group (18 mo: *n* = 8 CTRL, *n* = 7 VWR; 22 mo: *n* = 4 CTRL, *n* = 4 VWR). Representative photomicrographs of undecalcified midshaft cortical bone stained with (**C**) TRAP (for osteoclasts, red stain) and toluidine blue counterstain and (**D**) von Kossa (for mineral, black stain) and tetrachrome counterstain in 18 mo. Representative photomicrographs of undecalcified midshaft cortical bone stained with (**E**) TRAP and toluidine blue counterstain and (**F**) von Kossa and tetrachrome counterstain in 22 mo.
