# Supplementary material for: Body weight influences musculoskeletal adaptation to long-term voluntary wheel running during aging in female mice
Source: Aging (Albany NY). 2022 Nov 18;15(2):308–52. doi: 10.18632/aging.204390 (PMC9925690; doi:10.18632/aging.204390)
Supplement: Supplementary Tables [file aging-15-204390-s002.pdf]

SUPPLEMENTARY TABLES

Supplementary Table 1. Pearson correlation analysis of 18 mo old mice.

Parameters	Final body weight		Body weight change		BMAT/TA		Heart weight		Run distance	
	r	***p*-value**	***r***	***p*-value**	***r***	***p*-value**	***r***	***p*-value**	***r***	***p*-value**
Final Body Weight			0.85	6.02E-10	0.61	2.36E-04	0.69	1.03E-05		
BW Change	0.85	6.02E-10			0.65	6.21E-05	0.95	2.93E-17		
BMAT/TA	0.61	2.36E-04	0.65	6.21E-05			0.53	1.64E-03		
Heart Weight	0.69	1.03E-05	0.95	2.93E-17	0.53	1.64E-03				
Heart Weight/BW	−0.63	8.09E-05	−0.63	7.90E-05	−0.57	6.05E-04	−0.48	4.96E-03		
SOL_Area	0.51	1.10E-02	0.42	4.27E-02			0.44	3.02E-02		
SOL_Muscle Mass/BW			−0.46	6.74E-03						
EDL_Muscle Mass/BW	−0.81	7.99E-09	−0.80	3.06E-08	−0.46	8.80E-03	−0.73	1.52E-06		
EDL_Static CM									−0.54	2.44E-02
EDL_Contracted CM									−0.62	8.33E-03
EDL_Total Fiber Number	0.46	2.43E-02							0.62	3.00E-02
EDL_Type IIX			−0.52	8.76E-03			−0.57	3.50E-03		
EDL_Submax_ Fatigue	−0.46	8.79E-03	−0.43	1.67E-02						
EDL_Max_ Fatigue	−0.40	2.5E-02	−0.42	1.91E-02						
Empty Lacunae									−0.69	3.91E-02
Co.Th	0.45	9.34E-03	0.41	1.88E-02						
Co.Area	0.61	1.67E-04	0.60	2.31E-04	0.41	1.95E-02	0.52	2.07E-03		
Ultimate Load									−0.79	1.09E-02
Total Work to failure			−0.55	2.22E-02			−0.61	8.82E-03		
MOI	0.50	2.76E-02							0.69	3.79E-02

Pearson correlation analysis of 22 mo old mice.

Parameters	Final body weight		Body weight change		BMAT/TA		Heart weight		Run distance	
	r	***p*-value**	***r***	***p*-value**	***r***	***p*-value**	***r***	***p*-value**	***r***	***p*-value**
BW Change							−0.57	1.99E-02		
BMAT/TA							−0.53	3.54E-02		
Heart Weight/BW			−0.57	1.99E-02	−0.53	3.54E-02				
Total Lateral Heart Width					−0.65	6.60E-03	0.74	1.10E-03		
Left Ventricular Wall Thickness							0.59	1.54E-02		
SOL_Static CM	0.63	1.99E-02								
SOL_Contracted CM	0.64									
SOL_Max_Specific Force					0.52	4.05E-02				
SOL_Submax_Rate of Force Development	0.64									
SOL_Max_Zero Calcium	-0.64									
SOL_Submax_Zero Calcium	-0.68									
EDL_Static CM	0.69									
EDL_Contracted CM	0.82									
EDL_Max_Absolute Force	0.62									
EDL_Max_Specific Force	0.56									

EDL_Max_Rate of Relaxation	0.61									
EDL_Max_Rate of Force Development	0.60									
EDL_Submax_Rate of Force Development	0.55						−0.54	3.27E-02		
EDL_Max_Fatigue							−0.55	2.81E-02		
EDL_Max_Zero Calcium					0.58	1.87E-02				
EDL_Submax_Zero Calcium							−0.62	9.79E-03		
EDL_Max_Recovery from Fatigue			−0.58	1.91E-02	−0.53	3.43E-02				
EDL_Submax_Recovery from Fatigue									−0.83	1.11E-02
Tb. BV/TV	0.71									
Tb.Th					−0.67	4.56E-03				
Tb.N	0.72									
Tb.Sp	−0.62									
Tb.Conn.D	0.80									
Co.Th							−0.55	2.82E-02		
Nanoindentation_ Youngs's Modulus	0.85									
Nanoindentation_ Hardness	0.83		0.71	4.92E-02						

Supplementary Table 2. TUNEL assay of femurs from LBW/HBW mice with or without 6 mo endurance exercise.

GROUP	Category I (%)	Category II (%)	Category III (%)	Category VI (%)
		CTRL		
**CBW**	35.0 ± 11.1	30. 3 ± 5.7	27.4 ± 10.1	7.2 ± 5.5
	(23.7–55.2)	(23.9 - 38.7)	(12.2–42.1)	(0.7–16.9)
**LBW**	40.2 ± 10.3	29.8 ± 7.0	23.7 ± 12.9	6.3 ± 7.3
	(31.8–55.2)	(23.9–38.7)	(12.2–42.1)	(0.7–16.9)
**HBW**	29.9 ± 10.6	30.8 ± 5.2	31.1 ± 6.0	8.2 ± 3.9
	(23.7–45.7)	(26.3–36.5)	(24.9–39.4)	(2.8–12.1)
		VWR		
**CBW**	34.1 ± 17.0	28.5 ± 8.4	27.0 ± 6.6	10.4 ± 8.4
	(11.8–59.1)	(17.7–42.9)	(14.5–34.8)	(1.4–25.9)
**LBW**	25.0 ± 10.7	27.4 ± 9.4	34.5 ± 2.6	16.1 ± 8.3
	(11.8–36.0)	(17.7–37.0)	(29.1–34.8)	(6.4–25.9)
**HBW**	41.4 ± 18.6	29.4 ± 8.5	**23.5 ± 6.9** $^{p = 0.06}$	5.7 ± 5.5
	(17.0–59.1)	(22.4–42.9)	(14.5–31.3)	(1.4–13.2)

Data are mean ± SD (range). Abbreviations: CTRL: control group; VWR: voluntary wheel running group; CBW: Combined groups; LBW: Low body weight group; HBW: High body weight group; Category I: Live, Category II: Dying, Category III: Apoptotic, Category VI: Empty lacuna. No significant differences were found compared to the corresponding CTRL, but a decreased trend of category III was observed in HBW/VWR mice (p = 0.06).

Supplementary Table 3. Femoral mechanical properties in LBW/HBW mice with or without 6 mo endurance exercise.

GROUP	18 mo old	
	CTRL	VWR
**CBW**	$N = 8$	$N = 9$
**LBW**	$N = 4$	$N = 4$
**HBW**	$N = 4$	$N = 5$
**ULTIMATE LOAD (N)**		
**CBW**	12.7 ± 2.5	13.1 ± 3.4
	(10.2–16.9)	(7.0–17.8)
**LBW**	12.8 ± 3.0	13.6 ± 1.6
	(10.2–16.9)	(12.4–15.8)
**HBW**	12.6 ± 2.3	12.8 ± 4.6
	(10.2–14.9)	(7.0–17.8)
**ELASTIC STIFFNESS (N/mm)**		
**CBW**	57.3 ± 13.6	52.5 ± 14.5
	(43.0 – 82.4)	(19.9–67.4)
**LBW**	54.2 ± 11.3	50.1 ± 21.9
	(43.0–69.9)	(19.9–67.4)
**HBW**	60.4 ± 16.7	54.4 ± 7.1
	(46.4–82.4)	(45.5–63.0)
**ELASTIC MODULUS (GPa)**		
**CBW**	6.0 ± 1.8	5.6 ± 1.6
	(4.9–10.1)	(2.8–7.9)
**LBW**	6.4 ± 2.4	5.5 ± 2.2
	(5.0–10.1)	(2.8–7.9)
**HBW**	5.6 ± 0.9	5.7 ± 1.3
	(4.9–7.0)	(4.6–7.7)
**MOMENT OF INERTIA (mm^4)**		
**CBW**	0.16 ± 0.06	0.14 ± 0.02
	(0.10–0.27)	(0.11–0.17)
**LBW**	0.13 ± 0.02	0.13 ± 0.02
	(0.11–0.16)	(0.11–0.16)
**HBW**	0.18 ± 0.07	0.15 ± 0.02
	(0.10–0.27)	(0.13–0.17)

Data are mean ± SD (range). Abbreviations: CTRL: control group; VWR: voluntary wheel running group; CBW: Combined groups; LBW: Low body weight group; HBW: High body weight group; No significant differences were found compared to the corresponding CTRL.

Supplementary Table 4. Nanoindentation analysis of femurs from mice with or without long-term endurance exercise.

PARAMETER	18 mo old		22 mo old	
	CTRL ($N = 4$)	VWR ($N = 4$)	CTRL ($N = 4$)	VWR ($N = 4$)
Hardness (Gpa)	1.1 ± 0.6	1.6 ± 0.2	1.1 ± 0.6	0.4 ± 0.4
	(0.3–1.6)	(1.3–1.7)	(0.3–1.6)	(0.2–1.0)
Young's modulus (GPa)	21.7 ± 12.6	30.2 ± 3.4	21.8 ± 11.8	7.4 ± 5.6
	(4.7–33.6)	(25.4–33.4)	(4.7–30.3)	(4.3–15.8)

Abbreviations: CTRL: control group; VWR: voluntary wheel running group. No significant differences were found when compared to the corresponding CTRL.
